# Influence of Plant-Based Substrate Composition and Extraction Method on Accumulation of Bioactive Compounds in *Hericium erinaceus* (Bull.) Pers. Fruiting Bodies

**DOI:** 10.3390/molecules30153094

**Published:** 2025-07-24

**Authors:** Katarzyna Kała, Małgorzata Cicha-Jeleń, Katarzyna Sułkowska-Ziaja, Beata Ostachowicz, Ewa Węgrzynowicz, Jan Lazur, Agnieszka Szewczyk, Bożena Muszyńska

**Affiliations:** 1 Department of Medicinal Plant and Mushroom Biotechnology, Faculty of Pharmacy, Jagiellonian University Medical College, 9 Medyczna, 30-688 Kraków, Poland; malgorzata.cicha-jelen@uj.edu.pl (M.C.-J.); katarzyna.sulkowska-ziaja@uj.edu.pl (K.S.-Z.); ewa0.wegrzynowicz@student.uj.edu.pl (E.W.); jan.lazur@uj.edu.pl (J.L.); agnieszka.szewczyk@uj.edu.pl (A.S.); 2 Department of Medical Physics and Biophysics, Faculty of Physics and Applied Computer Science, AGH University of Krakow, 30 Adama Mickiewicza, 30-059 Kraków, Poland; beata.ostachowicz@fis.agh.edu.pl; 3 Doctoral School of Medical and Health Sciences, Jagiellonian University Medical College, 16 Św. Łazarza, 30-530 Kraków, Poland

**Keywords:** *Hericium erinaceus* (Bull.) Pers., plant-based cultivation substrates, natural products, antioxidant activity, bioactive compounds, bioelements, extraction methods, health improvement

## Abstract

The selection of plant-based substrates for mushroom cultivation is a key factor influencing their growth and metabolism. The aim of this study was to demonstrate, in an innovative approach, differences in the content of biologically active compounds, bioelements, and antioxidant properties of *Hericium erinaceus* (Bull.) Pers. cultivated on various plant-based substrates derived from waste materials, specifically hemp straw and beech sawdust. Another objective was to compare various extraction methods in terms of their impact on the concentration of these compounds. Elemental analysis was performed using the TXRF method, while bioactive constituents were determined using the DAD/UV RP-HPLC technique. The plant-based substrate and extraction method influenced the levels of obtained metabolites. Dual extraction with moderate ethanol concentrations was most effective for isolating key bioactive compounds from *H. erinaceus*—notably ergothioneine, lovastatin, L-phenylalanine, and ergosterol—while antioxidant activity did not correlate with the concentration of the solvent used. Although dual extracts enhanced certain antioxidants and metabolites, whole fruiting bodies contained higher levels of bioelements. Overall, fruiting bodies grown on beech sawdust had greater amounts of most bioactive compounds compared to those cultivated on hemp straw, emphasizing that both substrate choice and extraction method critically influence the mushroom’s bioactive profile and its potential health benefits.

## 1. Introduction

Currently, mushroom consumption is increasing due to their high nutritional value, desirable taste, and aroma [1,2]. Mushrooms provide the body with numerous nutrients, including easily digestible proteins, carbohydrates, bioelements, vitamins, fiber, and antioxidants [1,3]. They are characterized by a wide range of health-beneficial compounds, such as phenolic substances, sterols, alkaloids, lactones, terpenes, and ceramides [4]. Mushrooms are also an important source of essential bioelements [5].

The chemical composition of fruiting bodies is significantly influenced by the cultivation substrate, which acts not only as a physical support but also as a primary source of nutrients and biochemical precursors. The type, quality, and proportion of plant-based components in the substrate directly affect the metabolic pathways activated during mushroom growth, thereby modulating the synthesis and accumulation of bioactive compounds such as polysaccharides, terpenoids, phenolic compounds, and bioelements. This modulation of fungal metabolism is particularly relevant in the case of medicinal species, where the qualitative and quantitative profile of secondary metabolites directly influences their therapeutic potential [6,7]. Mushrooms possess the remarkable ability to convert agricultural waste [8]. Identifying substrates or substrate mixtures that yield larger and higher-quality crops is essential, as mushroom production technology is becoming increasingly important for recycling solid organic waste, enhancing nutritional value, and expanding medical applications [8].

Different species of mushrooms can effectively decompose substrates in various ways. Most mushrooms are adept at breaking down plant fibers derived from straw with relative ease. This adaptability makes them valuable agents in recycling agricultural by-products while enriching the substrates with nutrients beneficial for their growth and development [7]. Many mushroom species have been successfully cultivated on sawdust, which is often supplemented with wheat bran [7]. Mushroom cultivation on wood is widely practiced, particularly in Asia. Hardwood species such as birch, beech, and oak are commonly used in the form of logs or sawdust. Log cultivation typically involves inoculating spores into holes drilled into the logs, creating an ideal environment for fungal growth [7]. Straw is the most widely used substrate for mushroom cultivation due to its accessibility, ease of cutting, affordability, and simplicity in pasteurization [7]. One of the valuable species exhibiting the ability to grow on both straw and sawdust as cultivation substrates is *Hericium erinaceus* (Bull.) Pers.

*H. erinaceus* has attracted significant research interest as a functional food due to its numerous health-promoting properties [3,9]. Mature fruiting bodies of *H. erinaceus* can reach diameters of 10 to 30 cm and are distinguished by their appearance, characterized by long, pendulous spines resembling a lion’s mane that change color from white to yellow and eventually turn brown with age. The surface is soft and spongy, with a fibrous texture resulting from the dense arrangement of spines [9]. *H. erinaceus,* also known as “Houtou” or “Shishigashira” in China and “Yamabushitake” in Japan, has been used for centuries in Traditional Chinese Medicine (TCM), primarily for the treatment of gastrointestinal disorders such as gastritis and gastric ulcers, typically in the form of decoctions [3]. *H. erinaceus* is commercially consumed because of its delicious taste and high nutritional value [10]. Numerous therapeutic effects of this mushroom have been documented, including anti-inflammatory, antioxidant, anticancer, antibacterial, and neurotrophic activities [11,12,13]. Phytochemical studies have shown that its main components include erinacines, hericenones, and polysaccharides [11]. Hericenones and erinacines stimulate the synthesis of proteins essential for the proper functioning of the nervous system [6]. Recent studies have highlighted the beneficial effects of *H. erinaceus* fruiting bodies on depression, anxiety, and cognitive impairment [14]. *H. erinaceus* also exhibits protective effects against behavioral changes and histological alterations associated with Alzheimer’s disease through the modulation of oxidative and inflammatory pathways and the regulation of brain cellular stress [15].

The bioactive properties of *H. erinaceus* are strongly influenced not only by the composition of the cultivation substrate but also by extraction conditions, including the type and concentration of the solvent, as well as the extraction method employed—whether a single-step or a sequential dual water–alcohol extraction. These factors significantly affect both the yield and the composition of the extracted bioactive compounds. Consequently, optimizing extraction parameters is critical to maximize the recovery of active substances. Beyond solvent choice, the efficiency of the extraction process is also determined by other variables such as temperature, pH, extraction duration, storage conditions, solvent-to-solid ratio, and particle size [13,16].

The aim of this study was to obtain fruiting bodies of *H. erinaceus* cultivated on two different types of carefully selected plant-based substrates (hemp straw and beech sawdust) and assess whether a hemp-based substrate could serve as a superior and more cost-effective alternative to beech sawdust, offering a viable option within the framework of the circular economy by comparing their content of bioactive compounds relevant to health prevention. Beech sawdust is a commonly used and well-established substrate for the cultivation of polypore mushrooms. The incorporation of hemp straw into the substrate is associated with the recent increase in products derived from industrial hemp cultivation, which has, in turn, led to a rise in agricultural by-products. In line with the zero-waste principle, it is crucial to explore sustainable strategies for managing and minimizing these by-products, as they contain components beneficial to mushroom cultivation. This is particularly important because, both in basic research and in the food/agricultural industry, there are no clear guidelines regarding the optimal composition of cultivation substrates that influence fungal metabolism and the production of bioactive substances. A novelty of this study is that, to the best of our knowledge, we used hemp straw for the first time as an alternative substrate for the production of *H. erinaceus* fruiting bodies, which aligns with the sustainable management of by-products from hemp oil production and supports the principles of the circular economy by valorizing post-production by-products. Additionally, the study aimed to select optimal extraction methods and sample preparation procedures to obtain reliable and accurate results of bioactive compound content, which is crucial from the perspective of the consumer and the potential production of mushroom-based dietary supplements.

## 2. Results and Discussion

When analyzing the results obtained during the experiment, it is worth emphasizing that a wide range of substances was examined—from organic bioactive compounds, through bioelements, to the assessment of antioxidant potential, total phenolic content, and β-glucan levels. Analyzing the organic compound extraction results demonstrated that the preferred method for obtaining these substances is dual extraction (in the form of a paste), in which the first step is carried out using ethanol, followed by water extraction. This is a natural occurrence, as the dual extract represents a more concentrated form relative to powdered fruiting bodies [17]. The cultivation substrate A, composed of 55% hemp straw and 45% wheat bran, appears to be the more favorable medium, as particularly evident in the case of ergothioneine content (Table 1). The extraction method also influenced the content of this sulfur-based amino acid; dual extraction resulted in a higher ergothioneine content compared to one-step extraction with methanol or ethanol. For instance, the single-step extraction of *H. erinaceus* cultivated on hemp straw with ethanol yielded 250 mg/100 g d.w. of ergothioneine, whereas dual extraction produced concentrations ranging from 326 to 583 mg/100 g d.w. when the dual extracts were dissolved in ethanol alone. Due to the higher solubility of ergothioneine in water compared to methanol or ethanol, it is not surprising that the highest content of this compound was observed in the dual extract (using 70% of ethanol–HeDeHS70 dissolved in a 70:30 ethanol–water mixture which was 1558 mg/100 g d.w.). This highlights the rationale for performing two-step extractions, with a second step using water, to increase the yield of water-soluble substances from this type of material.

One of the greatest challenges in the mushroom cultivation industry is obtaining fruiting bodies of the highest quality, with high yields and enhanced resistance to stress [18]. Cultivation conditions are the primary factors influencing mycelial growth and the formation of fruiting bodies. The selection of plant-based substrates for the cultivation of medicinal mushrooms is therefore crucial [18]. Different species of mushrooms exhibit distinct preferences for growth substrates. For instance, shiitake and yellow oyster mushrooms thrive on straw and refined substrates. Reishi and maitake mushrooms perform better on sawdust and logs, whereas *Agaricus* spp. mushrooms are commonly cultivated using manure [7]. One study utilized coconut water to support the growth and enhance the yield, bioactive compounds, and antioxidant activity of *H. erinaceus* [19]. The use of a sawdust and rice mixture is an effective method for reducing production costs. For example, an optimal substrate for the production of *Trametes versicolor* was found to be a mixture consisting of 79% sawdust, 20% rice grains, and 1% calcium carbonate [18]. In another experiment, a substrate containing sawdust enriched with 1% wheat bran was used, which proved to be a suitable and cost-effective substrate for the cultivation of *Gandorema sinense* [18].

The development of efficient waste strategies is necessary to meet the increasing demand and consumption levels. Recently, the concept of a circular economy has gained more attention for its potential to achieve the fundamental goals of sustainable development [20]. Hemp straw is a residual biomass resource generated during the processing of hemp for applications in food, medicine, materials, or biofuels [20]. The use of plant-based additives in mushroom cultivation substrates is a commonly employed practice that aligns well with circular economy principles. Currently, mushroom cultivation seeks to replicate the fungi’s natural growth environments by using materials such as sawdust, which serve as both structural media and sources of nourishment for fungal mycelium, enabling its development and the formation of fruiting bodies. While traditional substrates contain the basic nutrients required by fungi, they are often insufficient as sole materials for producing high-quality mushrooms or stimulating the biosynthesis of valuable metabolites. Since the substrate is the most critical factor in mushroom development, the addition of supplementary nutrients is essential to enhance both yield and the synthesis of biologically active secondary metabolites. One such additive may be wheat bran, as applied in our experiment [21]. In one study, the impact of adding by-products derived from soybean meal production on the cultivation of *H. erinaceus* was evaluated. The results confirmed that *H. erinaceus* grown under these conditions exhibited higher mycelial growth and biological efficiency compared to mushrooms cultivated on control substrates. Furthermore, the bioactive compound content, such as triterpenoids and total phenolic content, in *H. erinaceus* grown on soybean meal was also higher than in the control sample. Additionally, *H. erinaceus* cultivated on soybean meal demonstrated enhanced 2,2-diphenyl-1-picrylhydrazyl radical (DPPH^•^) scavenging activity [21]. A common substrate component in commercial *H. erinaceus* cultivation is sawdust from various hardwood species [22]. The use of wood chips from coniferous trees, specifically from the *Pinus* species, is also known for cultivation purposes. For *H. erinaceus* cultivation, materials such as wheat and rice straw, as well as corn cobs, are commonly used. Additionally, substrates are often supplemented with various additives, including cereal bran or grains, by-products from sugarcane production, bone meal, and soy meal, to enhance both the yield and quality of the crop [22]. Some researchers have identified beech sawdust enriched with a 20% addition of wheat bran as the optimal substrate for *H. erinaceus* cultivation, which is somewhat similar to our approach, where wheat bran was also used as a plant-based additive to enhance substrate composition [22].

In the following section, the influence of the substrates used in the experiment, along with the applied extraction methods, on the content of specific bioactive compounds and the antioxidant potential in *H. erinaceus* is analyzed.

### 2.1. Ergothioneine

Ergothioneine, a non-protein amino acid, is a thioimidazole derivative of histidine, found in large quantities in mushrooms. Due to its health-promoting effects, it is referred to as the “longevity vitamin”. It is essential for humans and can only be obtained through dietary intake. Ergothioneine exhibits antioxidant activity by scavenging reactive oxygen species and activating antioxidant enzymes [23,24]. Cells with lower levels of this compound are more susceptible to the effects of oxidative damage, including lipid peroxidation and defects in the oxidation of proteins or DNA [24].

The conducted study revealed a significant impact of the extraction solvent on the quantity of the extracted compound. A representative chromatogram of the determined ergothioneine is presented in Figure 1. The chromatograms for ergothioneine are presented in the Supplementary Data, Figures S1–S16.

**Figure 2:**
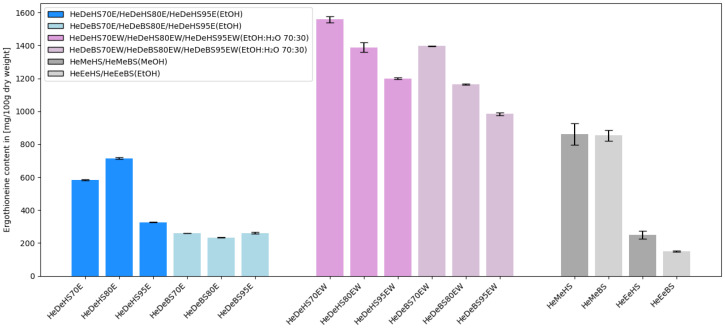
Comparison of ergothioneine content in various types of extracts obtained from *Hericium erinaceus* (He—*Hericium erinaceus* fruiting bodies; De—dual extract; HS—hemp straw substrate, BS—beech sawdust substrate; 70/80/95—ethanol concentration used for dual extract preparation; E/EW—dual extract dissolved in HPLC-grade 96% ethanol (E) or 70:30 ethanol–water mixture (EW); Me—liquid methanolic extract; Ee—liquid methanolic extract; detailed description can be found below Table 1).

Compared to ethanol, methanol exhibited markedly higher efficiency as an extraction solvent in the case of conventional liquid extracts obtained using an ultrasonic bath (Figure 2).

It is important to note that the method of preparing the sample from the dual extract—specifically, dissolving it in a 70:30 ethanol-to-water mixture—significantly influenced the determination of ergothioneine content (984 to 1558 mg/100 g dry weight (d.w.)) (Table 1). The high ergothioneine content found in *H. erinaceus* samples is consistent with the results of previous experiments. Analysis of the ergothioneine content results demonstrated that the preferred method for obtaining this compound is dual extraction (resulting in a paste-like extract), in which the first step is carried out using 70% ethanol, followed by aqueous extraction. Notably, the highest ergothioneine content was obtained in the paste derived from fruiting bodies of *H. erinaceus* cultivated on a substrate composed of 55% hemp straw and 45% wheat bran and subjected to this specific extraction scheme—the ergothioneine content reached as much as 1558 mg/100 g d.w. The use of ethanol solutions at higher concentrations (80% and 95%) resulted in a marked decrease in extraction efficiency—lower levels of ergothioneine were detected in mushroom material obtained from both hemp straw-based and beech sawdust-based substrates, confirming that a lower ethanol concentration in the first extraction step is more optimal for isolating this valuable compound (Figure 2). Previous studies have reported ergothioneine levels in mushroom fruiting bodies ranging from approximately 40 to 220 mg/100 g d.w., which is comparable to the values obtained in our study using conventional liquid extracts, confirming *H. erinaceus* as a competitive natural source of this antioxidant [6,25]. In other edible mushroom species, particularly in oyster mushrooms (*Pleurotus* spp.), nearly 700 mg of this compound has been detected, which further highlights the nutritional importance of mushrooms in the human diet [26].

### 2.2. Lovastatin

Lovastatin is one of the statins that significantly lowers cholesterol levels and acts as an inhibitor of the key enzyme regulating cholesterol production (3-hydroxy-3-methylglutaryl-coenzyme A reductase) [27]. Lovastatin was the first statin approved by the U.S. Food and Drug Administration (FDA) as a hypocholesterolemic drug [27]. By reducing total cholesterol and low-density lipoproteins (LDL), lovastatin is effective in preventing cardiovascular diseases, including atherosclerosis. It also exhibits antioxidant and anti-inflammatory properties [28]. 

*Aspergillus terreus* is an example of a fungus that produces significant amounts of lovastatin, reaching up to 867 mg/100 g d.w., which is considerably higher compared to the lovastatin content in edible mushrooms [6]. In previous studies, the estimated lovastatin content in *H. erinaceus* mycelium was found to be 18.7 mg/100 g d.w. [6]. The highest lovastatin content was found in paste-form dual extracts obtained from fruiting bodies cultivated on a substrate composed mainly of beech sawdust, ranging from 3.15 to 3.27 mg/100 g d.w. (Table 1). Although no statistically significant differences were observed in the lovastatin content among the individual dual extracts prepared in the form of pastes, differences were noted between the dual extracts and the classical liquid methanolic and ethanolic extracts. The dual extracts in the most cases contained higher amounts of lovastatin compared to single-step extraction with methanol or ethanol (Table 1). The chromatograms for lovastatin are presented in the Supplementary Materials, Figures S17–S32.

### 2.3. Serotonin, L-Tryptophan, 5-Hydroxy-L-Tryptophan

Serotonin serves as the most important endogenous neurotransmitter in the human body and can also exhibit antioxidant activity. However, exogenous serotonin is of less significance compared to its endogenous counterpart [24]. One of the ways to increase endogenous serotonin levels is by incorporating foods containing another indole derivative, such as L-tryptophan—a direct precursor of serotonin in the central nervous system—into the diet [24]. Serotonin levels may also be supported by dietary sources of 5-hydroxy-L-tryptophan (5-HTP), a natural intermediate in serotonin biosynthesis involved in the regulation of mood, sleep, and appetite [29].

In our conducted experiment, the highest level of L-tryptophan was detected in samples prepared from methanolic extracts of *H. erinaceus* cultivated on beech sawdust (17.0 mg/100 g d.w.). Samples obtained in dual extracts form contained very low levels of L-tryptophan (quantified only in case of dual extract from *H. erinaceus* fruting bodies extracted with 95% of ethanol) or were even below the detection threshold (Table 1). The chromatograms for indole compounds are presented in the Supplementary Materials, Figures S33–S36.

### 2.4. L-Phenylalanine

Phenylalanine is an essential aromatic exogenous amino acid necessary for proper organism function and must be obtained through the diet. It plays a crucial role as a precursor in the biosynthesis of other biologically active compounds—including tyrosine, dopamine, norepinephrine, and epinephrine—thereby directly linking it to the functioning of the nervous system and processes regulating mood, concentration, and stress response. Dietary sources of phenylalanine include animal-derived proteins (meat, eggs, and dairy), plant-based proteins (legume seeds, nuts, and soy), as well as edible mushrooms [6].

Analysis of the extraction results showed that the preferred method for obtaining this compound is a dual extraction, where the first step involves 95% ethanol, followed by an aqueous extraction. The highest content was found in *H. erinaceus* fruiting bodies grown on a substrate composed of 55% beech sawdust and 45% wheat bran, subjected to this extraction procedure—the amount reached 531 mg/100 g d.w. However, the quantification results display considerable variability, which is fully consistent with previously conducted analyses [6,28]. The chromatograms for L-phenylalanine are presented in the Supplementary Materials, Figures S37–S52.

### 2.5. Ergosterol and Tocopherol

Ergosterol exhibits anti-inflammatory, anti-tyrosinase, and anticancer properties. It is the most prevalent sterol found in fungal cell membranes and also serves as a precursor to vitamin D_2_ [30]. Vitamin D is well-known for supporting bone health and calcium homeostasis, as well as exerting positive effects on immune function and cellular physiology [30]. *H. erinaceus* is rich in a group of metabolites with antioxidant, anti-inflammatory, and anti-osteoporotic properties [6,7].

**Figure 3:**
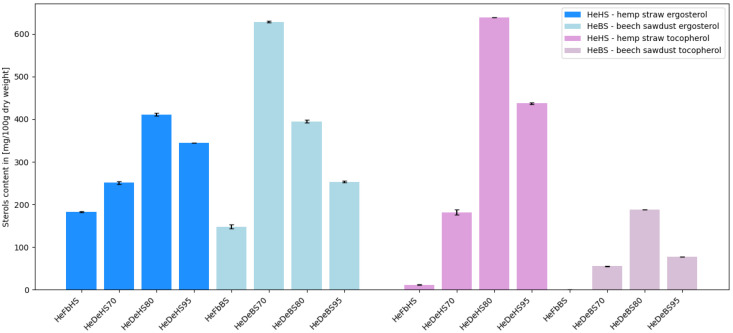
Comparison of sterol content in various types of extracts obtained from *Hericium erinaceus* (He—*Hericium erinaceus*; Fb—fruiting bodies; De—dual extract; HS—hemp straw substrate, BS—beech sawdust substrate; 70/80/95—ethanol concentration used for dual extract preparation; detailed description can be found below Table 2).

The highest amount of vitamin D precursor, ergosterol, was found in the *H. erinaceus* dual extract prepared using 70% ethanol and water, obtained from fruiting bodies cultivated on a substrate composed of beech sawdust and wheat bran (628 mg/100 g d.w.) (Figure 3).

**Table 2:** The content of bioelements in *Hericium erinaceus* fruiting bodies cultivated on substrates supplemented with hemp straw and beech sawdust in dual water–ethanol extracts in paste form, as well as in traditionally prepared liquid methanolic and ethanolic extracts [expressed in mg per 100 g of dry fruiting body or per 100 g of dual extract].

	HeFbHS	HeFbBS	HeDeHS70	HeDeHS80	HeDeHS95	HeDeBS70	HeDeBS80	HeDeBS95	HeMeHS	HeMeBS	HeEeHS	HeEeBS
K	3785 ± 70 ^b^	4129 ± 221 ^a^	1028 ± 42 ^d^	606 ± 51 ^e^	1263 ± 71 ^d^	1692 ± 167 ^c^	1588 ± 34 ^c^	1297 ± 59 ^d^	109 ± 7 ^f^	1075 ± 88 ^d^	60.4 ± 0.5 ^f^	77.7 ± 4.4 ^f^
Ca	12.9 ± 1.8 ^c^	18.1 ± 0.4 ^b^	*	*	*	*	*	*	14.3 ± 0.6 ^c^	26.0 ± 0.4 ^a^	8.43 ± 0.94 ^d^	2.72 ± 0.29 ^e^
Zn	9.67 ± 0.04 ^a^	7.41 ± 0.07 ^b^	0.588 ± 0.004 ^e^	0.310 ± 0.003 ^f^	0.707 ± 0.006 ^d^	0.868 ± 0.007 ^c^	0.678 ± 0.004 ^d^	0.851 ± 0.003 ^c^	0.107 ± 0.030 ^g^	0.106 ± 0.022 ^g^	0.029 ± 0.002 ^h^	0.012 ± 0.002 ^h^
Rb	2.19 ± 0.04 ^a^	1.92 ± 0.01 ^b^	0.291 ± 0.003 ^g^	0.187 ± 0.005 ^h^	0.383 ± 0.006 ^f^	0.826 ± 0.018 ^c^	0.799 ± 0.006 ^c^	0.669 ± 0.019 ^d^	0.086 ± 0.003 ^i^	0.452 ± 0.039 ^e^	0.018 ± 0.001 ^j^	0.035 ± 0.004 ^i, j^
Fe	9.87 ± 0.05 ^a^	8.34 ± 0.06 ^b^	1.42 ± 0.01 ^f^	0.919 ± 0.013 ^g^	2.08 ± 0.05 ^e^	3.34 ± 0.49 ^c^	3.36 ± 0.02 ^c^	2.84 ± 0.05 ^d^	0.365 ± 0.005 ^h^	0.186 ± 0.201 ^h^	0.198 ± 0.004 ^h^	0.075 ± 0.005 ^h^
Cu	6.73 ± 0.03 ^a^	3.98 ± 0.03 ^b^	0.416 ± 0.002 ^f^	0.366 ± 0.003 ^f^	0.868 ± 0.019 ^e^	0.962 ± 0.029 ^d^	0.973 ± 0.070 ^d^	1.135 ± 0.017 ^c^	0.023 ± 0.008 ^h^	0.287 ± 0.013 ^g^	0.008 ± 0.001 ^h^	0.022 ± 0.003 ^h^
Ni	0.279 ± 0.024 ^b^	0.472 ± 0.019 ^a^	0.035 ± 0.003 ^f^	0.035 ± 0.004 ^f^	0.037 ± 0.007 ^f^	0.049 ± 0.004 ^e, f^	0.026 ± 0.003 ^f^	0.035 ± 0.003 ^f^	0.162 ± 0.014 ^c^	0.089 ± 0.021 ^d^	0.083 ± 0.003 ^d, e^	0.056 ± 0.015 ^d, e, f^
Sr	0.098 ± 0.021 ^a^	0.128 ± 0.015 ^a^	0.026 ± 0.002 ^b, c^	0.032 ± 0.008 ^b, c^	0.031 ± 0.002 ^b, c^	0.035 ± 0.004 ^b, c^	0.045 ± 0.019 ^b^	0.029 ± 0.006 ^b, c^	0.033 ± 0.010 ^b, c^	0.033 ± 0.014 ^b, c^	0.017 ± 0.005 ^b, c^	0.006 ± 0.001 ^c^
Se	0.018 ± 0.003 ^b^	0.016 ± 0.001 ^b, c^	0.006 ± 0.001 ^d, e, f^	0.004 ± 0.001 ^e, f^	0.006 ± 0.001 ^d, e, f^	0.011 ± 0.001 ^c, d^	0.010 ± 0.001 ^c, d, e^	0.009 ± 0.002 ^d, e^	0.081 ± 0.010 ^a^	0.005 ± 0.001 ^d, e, f^	0.001 ± 0.001 ^f^	0.002 ± 0.001 ^f^
Mn	1.59 ± 0.01 ^b^	1.90 ± 0.01 ^a^	0.264 ± 0.001 ^g^	0.158 ± 0.001 ^h^	0.387 ± 0.005 ^f^	0.578 ± 0.006 ^d^	0.675 ± 0.006 ^c^	0.471 ± 0.005 ^e^	0.005 ± 0.001 ^j^	0.033 ± 0.008 ^i^	0.003 ± 0.001 ^j^	0.003 ± 0.001 ^j^
Br	0.077 ± 0.005 ^c, d^	0.062 ± 0.002 ^d^	0.012 ± 0.001 ^g^	0.011 ± 0.001 ^g^	0.016 ± 0.001 ^f, g^	0.043 ± 0.006 ^e^	0.029 ± 0.001 ^e, f^	0.017 ± 0.001 ^f, g^	0.165 ± 0.008 ^a^	0.039 ± 0.007 ^e^	0.083 ± 0.001 ^c^	0.102 ± 0.014 ^b^
Ti	0.598 ± 0.033 ^b^	0.690 ± 0.058 ^a^	0.073 ± 0.006 ^c, d, e^	0.107 ± 0.002 ^c, d^	0.079 ± 0.007 ^c, d, e^	0.131 ± 0.057 ^c^	0.091 ± 0.010 ^c, d, e^	0.077 ± 0.010 ^c, d, e^	0.097 ± 0.007 ^c, d, e^	0.030 ± 0.010 ^e^	0.046 ± 0.008 ^d, e^	0.024 ± 0.002 ^e^
Cr	0.113 ± 0.012 ^a^	0.103 ± 0.010 ^a^	0.028 ± 0.002 ^e, f^	0.012 ± 0.001 ^f, g^	0.035 ± 0.004 ^d, e^	0.071 ± 0.005 ^b^	0.065 ± 0.004 ^b, c^	0.049 ± 0.009 ^c, d^	0.021 ± 0.010 ^e, f, g^	0.020 ± 0.005 ^e, f, g^	0.008 ± 0.001 ^g^	0.006 ± 0.001 ^g^

*n* = 3; *—sample matrix interference; **HeFbHS**—*Hericium erinaceus* fruiting bodies grown on hemp straw substrate; **HeFbBS**—*Hericium erinaceus* fruiting bodies grown on beech sawdust substrate; **HeDeHS70**—dual extract (using 70% ethanol solution) prepared from *Hericium erinaceus* fruiting bodies cultivated on a hemp straw substrate; **HeDeHS80**—dual extract (using 80% ethanol solution) prepared from *Hericium erinaceus* fruiting bodies cultivated on a hemp straw substrate; **HeDeHS95**—dual extract (using 95% ethanol solution) prepared from *Hericium erinaceus* fruiting bodies cultivated on a hemp straw substrate; **HeDeBS70**—dual extract (using 70% ethanol solution) prepared from *Hericium erinaceus* fruiting bodies cultivated on a beech sawdust substrate; **HeDeBS80**—dual extract (using 80% ethanol solution) prepared from *Hericium erinaceus* fruiting bodies cultivated on a beech sawdust substrate; **HeDeBS95**—dual extract (using 95% ethanol solution) prepared from *Hericium erinaceus* fruiting bodies cultivated on a beech sawdust substrate; **HeMeHS**—liquid methanolic extract obtained from *Hericium erinaceus* fruiting bodies cultivated on a hemp straw substrate; **HeMeBS**—liquid methanolic extract obtained from *Hericium erinaceus* fruiting bodies cultivated on a beech sawdust substrate; **HeEeHS**—liquid ethanolic extract obtained from *Hericium erinaceus* fruiting bodies cultivated on a hemp straw substrate; **HeEeBS**—liquid ethanolic extract obtained from *Hericium erinaceus* fruiting bodies cultivated on a beech sawdust substrate; the letters next to values represent Tukey’s HSD post hoc results (*p* < 0.05) and compare the results on the same line (significance difference for means within the same row).

In the dual extracts, as in the case of most of the analyzed bioactive compounds, significantly higher ergosterol content was determined compared to the powdered fruiting bodies. In previous studies, other authors reported an ergosterol content of approximately 252 mg/100 g d.w. in *H. erinaceus* fruiting body extracts, which is higher than the values determined in our analysis of fruiting bodies (183 mg/100 g d.w. on hemp straw substrate and 148 mg/100 g d.w. on beech sawdust substrate) [30,31]. The chromatograms for sterols are presented in the Supplementary Materials, Figures S53–S60.

In the past, α-tocopherol was considered the most biologically active form of vitamin E in humans, and it has been demonstrated to exhibit the highest biological activity [32]. Tocopherols are methyl-substituted hydroxychromans consisting of a polar chromanol ring and a nonpolar phytyl side chain. Four tocopherol vitamers (α-, β-, γ-, and δ-tocopherol) are distinguished based on the number and position of methyl groups on their chromanol ring [33].

A high intake of vitamin E may reduce the risk of several chronic diseases associated with oxidative damage [33]. Based on the obtained results, it can be concluded that dual extracts derived from the fruiting bodies of *H. erinaceus*, cultivated on hemp straw with the addition of wheat bran, exhibited a higher tocopherol content (Figure 3).

### 2.6. Bioelements

Bioelements are essential for proper human body function, playing key roles in metabolic, structural, and regulatory processes. The content of bioelements in the mycelium is dependent on its ability to absorb them from the substrate. Identified bioelements are essential for the proper functioning of the human body, which is directly related to the potential health-promoting effects of mushrooms. Both deficiencies and excesses can cause serious health issues, so adequate intake through diet—such as through edible mushrooms that accumulate many bioelements or through supplements derived from them—is vital for health maintenance and disease prevention [6]. Zinc is one of the essential trace elements in living organisms, playing a vital role in cellular function and human metabolism. A deficiency in Zn can lead to oxidative damage in the human body by inhibiting the formation of reactive oxygen species (ROS) [34].

The content of individual micro- and macroelements in the analyzed samples is summarized in Table 2. The fruiting bodies of *H. erinaceus* grown on a substrate composed of 55% hemp straw and 45% wheat bran proved to be the best source of selenium, zinc, and iron. The Se content measured in the analyzed fruiting bodies may support the diet and help prevent deficiencies, similarly to Zn and Fe, which are also commonly found in commercially available supplements. The maximum Se content (18 µg/100 g d.w.)—as well as the levels of iron and zinc (around 10 mg/100 g d.w.)—are consistent with the data reported in the scientific literature [35]. Significantly lower contents of the analyzed elements were found in dual extracts compared to the fruiting bodies. Notably, in paste-type preparations (dual extracts), higher microelement contents were observed in products obtained from fruiting bodies cultivated on beech sawdust substrate (55% beech sawdust and 45% wheat bran). Nevertheless, consuming whole fruiting bodies remains a more advantageous form of supplementation due to their higher concentrations of bioelements (unlike, for example, ergothioneine or antioxidant potential) (Table 1, Table 2 and Table 3).

### 2.7. β-Glucans

Mushrooms owe their sensory properties, nutritional value, and health benefits to bioactive compounds, particularly β-glucans [4]. Glucans exhibit bioactive properties, such as immunomodulatory, anticancer, antiviral, and hepatoprotective effects [2,4]. These effects contribute to reducing the risk of various diseases, including neurodegenerative disorders. β-glucans belong to the group of prebiotics, which significantly influence the functioning of gut microbiome, thereby exhibiting neuroprotective effects [23].

The primary structural feature of mushroom β-glucans is a β-1,3-D-glucan main chain with single D-glucosyl residues linked to β-1,3 along this main chain [4]. β-glucans are not synthesized by the human body and are recognized by the immune system, triggering both adaptive and innate immune responses [4,36]. The methods previously used for the extraction of *H. erinaceus* polysaccharides primarily include water or alkaline extraction, additionally enhanced by ultrasound, microwaves, or enzymes [9]. It has been demonstrated that ultrasonic treatment accelerates the dissolution of polysaccharide molecules in the extractant by breaking down cell walls [9]. Due to the multilayered structure of the cell wall of *H. erinaceus* fruiting bodies, β-glucans often remain insoluble in water during water extraction [9]. The highest β-glucan content was observed in dual extracts obtained using 95% ethanol as the solvent, with the highest values recorded in samples derived from fruiting bodies cultivated on hemp straw supplemented with wheat bran (Table 3). The determined β-glucan content in this sample was 0.391 g/100 g of extract. The obtained results indicate that the 80% ethanol solution was the least effective solvent for extracting β-glucans in dual extracts. Similarly, powdered fruiting bodies also showed low β-glucan content, suggesting limited extractability in both forms (Table 3).

### 2.8. Antioxidant Activity

Free radicals are reactive molecules formed as by-products of metabolic processes. Elevated concentrations of these molecules can lead to cellular damage. Oxidative stress plays a significant role in the pathogenesis of various diseases, including cancer, central nervous system disorders, and cardiovascular diseases. The role of antioxidants is to reduce or inhibit the action of free radicals produced during chemical reactions and metabolic events within the body [13,34,37]. To defend against the harmful effects of radicals, living organisms utilize both exogenous and endogenous antioxidants. Antioxidants play a critical metabolic role. The investigation of antioxidant activity in substances extracted from food has been a subject of scientific research for many years [38]. Although some chemically synthesized antioxidants can effectively mitigate oxidative damage, they may also pose potential risks of liver damage in humans [39].

*H. erinaceus* is rich in various active compounds with antioxidant properties. The polysaccharides from *H. erinaceus* perform well in both enzymatic and non-enzymatic oxidation processes [9]. Polysaccharides from this species can effectively reduce the production of reactive oxygen species (ROS) and regulate enzyme activities, such as superoxide dismutase, thereby alleviating tissue and organ damage caused by oxidative stress [9]. In one study, a polysaccharide was extracted from *H. erinaceus* cultivated on tofu whey. Mushrooms grown under these conditions exhibited strong hydroxyl radical scavenging activity, DPPH^•^ radical scavenging activity, and iron-chelating ability [39].

Available methods for measuring antioxidant activity can be classified based on their mechanism of action, in which the applied compound halts chain-breaking reactions. They can be divided into two groups: hydrogen atom transfer (HAT) reactions and single-electron transfer (SET) reactions, which involve the reduction in compounds through electron transfers from antioxidants [37,40]. ABTS and DPPH methods are among the most popular techniques for determining antioxidant activity [38].

In the case of *H. erinaceus*, better results were obtained with a substrate composed of 55% beech sawdust and 45% wheat bran. The fruiting bodies, and subsequently the pastes (dual extracts) derived from them, exhibited higher antioxidant potential in most of the analyzed samples (Table 3). No significant effect of ethanol concentration used in the extraction process was observed in the assessment of antioxidant activity—70%, 80%, and 95% ethanol solutions yielded comparable results in both the DPPH and ABTS assays. In contrast, the results for liquid methanolic and ethanolic extracts obtained from fruiting bodies were significantly lower, which is understandable given that a proportionally smaller amount of fruiting bodies was used in the extraction process, resulting in a less concentrated extract (Table 3).

### 2.9. Total Phenolic Compound Content

The most reliable results were observed for dual extracts dissolved in a 70:30 ethanol–water mixture. It is evident that the sample preparation method plays a crucial role and ultimately determines the total phenolic content measured. In the case of liquid methanolic and ethanolic extracts, methanol proved to be the more effective solvent. Analysis of the results indicated that fruiting bodies cultivated on substrates predominantly composed of beech sawdust exhibited higher total phenolic compound contents (Table 3). Previously, the authors reported total phenols at the level of several to a dozen mg GAE/g d.w., which is consistent with the ethanolic liquid extracts obtained in the current experiment; however, significantly higher total phenol contents were detected in the methanolic extracts and in all dual extracts [41,42].

From a biochemical standpoint, the observed differences in the accumulation of specific metabolites such as ergothioneine, lovastatin, and L-phenylalanine suggest that the composition of the cultivation substrate can modulate fungal metabolic pathways by influencing the availability of biochemical precursors and environmental stimuli. Hemp straw, rich in lignocellulosic compounds, may promote oxidative stress-related responses, thereby enhancing the synthesis of antioxidant molecules via thiol- and shikimate-dependent pathways. Moreover, dual extraction allows access to both hydrophilic and lipophilic metabolite fractions, reflecting the diverse solubility profiles of fungal bioactives and improving the recovery of their biochemical potential. These findings are consistent with the current knowledge on the metabolic plasticity of *H. erinaceus* and other medicinal mushrooms cultivated on alternative substrates [43].

## 3. Materials and Methods

### 3.1. Obtaining of Fruiting Bodies

Fruiting bodies cultivated on a farm in southern Poland (Osiek 49°57′03″ N, 19°15′50″ E; 262 m above sea level) in April/May 2024 were used as the research material. The botanical identification was conducted based on an analysis of morphological characteristics using the MycoKey 4.1 key [44]. Fruiting bodies of the species *H. erinaceus* were obtained from two types of substrates: one composed of 55% hemp straw and 45% wheat bran, and the other consisting of 55% beech sawdust and 45% wheat bran. Both cultivation substrates were commercially analyzed by Eurofins Katowice (Poland) for the presence of potentially harmful substances, including heavy metals (cadmium, lead, arsenic, and mercury), polycyclic aromatic hydrocarbons (benzo(α)anthracene, benzo(α)pyrene, benzo(α)fluoranthene, chrysene, and total polycyclic aromatic hydrocarbons), and selected pesticides (piperonyl butoxide, cypermethrin, glyphosate, and aminomethylphosphonic acid). The results confirmed that the substrate components were safe and suitable for the cultivation of edible mushrooms. These substrates were selected due to their high availability, sustainability, and proven suitability for supporting the growth and development of medicinal mushrooms, providing both structural support and essential nutrients for fruiting body formation. The cultivation of *H. erinaceus* fruiting bodies began with the use of liquid mycelial cultures, which were transferred onto sterilized and hydrated wheat grains. These grains served as a grain spawn and were placed in Unicorn Type 3 bags (Plano, Tx, USA) equipped with 0.2 μm microfilters. The production substrate was prepared from a volumetric mixture of hemp straw or beech sawdust and wheat bran, moistened to achieve 55% relative humidity. After thorough mixing, 2.5 kg of the substrate was loaded into filter bags and sterilized via autoclaving (121 °C, 1 atm, and 2 h). Once cooled, the substrate was inoculated with the grain-based mycelium and incubated in darkness at 23 ± 2 °C until full colonization. Subsequently, the bags were opened and transferred to a fruiting chamber with a constant temperature of 20 °C and a photoperiodic lighting regime (600 lux). Fruiting occurred in two flushes, observed approximately 2 and 4 weeks after transfer. Representative fruiting bodies from the first flush were selected for analysis. Samples of species were deposited in the Department of Medicinal Plant and Mushroom Biotechnology at the Jagiellonian University Medical College under the accession number HeKB0424(1) for *H. erinaceus* fruiting bodies cultivated on beech sawdust or HeKB0424(2) for *H. erinaceus* fruiting bodies cultivated on hemp straw.

### 3.2. Description of Drying and Extraction—Dual Extract Preparation

A food dehydrator (Royal Catering RCDA-1350/100S, Berlin, Germany) was used for drying the mushroom material (fruiting bodies), while grinding was carried out using a knife mill (Royal Catering RCMZ-1000, Berlin, Germany). Ultrasonic-Assisted Extraction (UAE) was performed using an ultrasonic homogenizer from Hielscher (Hielscher UP400St, Berlin, Germany) equipped with a cascatrode (ø 22 mm, length 204 mm) with a power of 400 W, a frequency of 24 kHz, and adjustable amplitude. The separation of biomass from the aqueous extract was conducted using a laboratory centrifuge (OHAUS Frontier FC5916, Nänikon, Switzerland). For extraction with ethanol solutions, biomass separation from the filtrate was carried out via vacuum filtration (filter pore size: 10 μm, KNF N938 LABOPORT pump, Lublin, Poland), while concentration was performed using a rotary evaporator.

### 3.3. Ultrasound-Assisted Extraction for Dual Extracts Preparation

An ultrasonic extractor was employed to conduct a solvent extraction cycle under the following constant conditions: amplitude at 100% and process duration of 30 min. A sample of 50 g was mixed with an aqueous-ethanol extraction solution containing 70%, 80%, and 95% ethanol (*v/v*) at a mass ratio of 1:10 and subjected to ultrasound treatment. The extraction was carried out at room temperature, with continuous monitoring of temperature increase, ensuring it remained below 45 °C through the use of an ice bath. A total of 500 mL of solvent was used for the extraction. Biomass separation from the extraction liquid was performed via vacuum filtration. The filtrate was subsequently concentrated by distilling off solvents using a rotary evaporator. To maximize the recovery of bioactive substances from the extracted fruiting bodies and mitigate solvent volume limitations, the entire process was repeated twice.

In the second stage, the residues from the ethanol extraction were further extracted. The sample was mixed with distilled water at a mass ratio of 1:10 and subjected to ultrasonic treatment under fixed conditions: amplitude at 100% and process duration of 30 min. The temperature was monitored using a sensor and maintained below 45 °C via an ice bath. Separation of biomass from the extraction liquid was conducted using a laboratory centrifuge, which applied centrifugal force for mechanical separation of solids from liquids. The filtrate was subsequently combined with the ethanol fraction and concentrated by distilling off solvents using a rotary evaporator.

Then, water–ethanol extracts previously prepared from the mushroom material from both substrates were used in the study. The ratios of solvents used were as follows: 70% ethanol–30% water; 80% ethanol–20% water; and 95% ethanol–5% water. For further analysis, the obtained dual extracts were dissolved in HPLC-grade ethanol and a mixture of ethanol and water (70:30) to ensure complete solubilization of the test material and to prevent result distortion due to the limited solubility of the dual extracts. To conduct the analyses, 0.5 g of prepared dual extract was dissolved in 4 mL of solvent. 

### 3.4. Ultrasound-Assisted Extraction for Methanolic and Ethanolic Extracts Preparation

The second form of research material consisted of fruiting bodies that underwent lyophilization (Freezone 4.5 lyophilizer, Labconco, Kansas City, MO, USA), followed by homogenization using an agate mortar and an EGK analytical grinder (Rommelsbacher, Dinkelsbühl, Germany). Next, 4 g of each sample was weighed and transferred into 150 mL glass beakers in the case of methanolic extracts, and 2 g in the case of ethanolic extracts. To each beaker, 120 mL of analytically pure methanol was added in the case of methanolic extracts, and 60 mL of ethanol in the case of ethanolic extracts until the supernatant was discolored (~1080 mL and 540 mL of each solvent, respectively). The extraction process was carried out using an ultrasound. For this purpose, the beakers containing the biomass and ethanol were placed in an ultrasonic bath (Polsonic, Warsaw, Poland) for 20 min. After this time, the extracts were filtered through fluted filter paper into 300 mL crystallizers and left at room temperature for ethanol evaporation. The remaining biomass in the beakers was treated with another part of analytically pure methanol and ethanol, and the extraction process was repeated. The extracts were filtered again through fluted filter paper into crystallizers from the first stage. This process was repeated nine times. After complete solvent evaporation from the crystallizers—either at room temperature or using a vacuum evaporator—the dry residue was quantitatively dissolved in HPLC-grade methanol or ethanol. The prepared extracts were stored in a refrigerator. For chromatographic analyses, 2 mL of each extract was filtered through 0.22 µm PTFE syringe filters into glass vials. 

### 3.5. Analysis of the Contents of Bioactive Compounds

The chromatographic analysis of organic compounds was conducted using the reversed-phase high-performance liquid chromatography (RP-HPLC) method, based on standard calibration curves, assuming a linear correlation between the area under the curve (AUC) and the concentration of reference substances. The total content of bioactive compounds was expressed in milligrams per 100 g of dual extract or fruiting bodies. Each analyzed sample was examined in three independent replicates.

#### 3.5.1. Analysis of Ergothioneine

The analysis of ergothioneine content was performed using the RP-HPLC method based on the procedure described by Zhou et al. [45]. Extracts (methanolic, ethanolic, and dual extracts dissolved in various solvents) were subjected to chromatographic analysis. A Merck Hitachi LaChrom liquid chromatography system equipped with a UV L-7400 detector (Merck Hitachi, Tokyo, Japan), RP-18e 4 × 250 mm column (Purospher^®^, particle size 5 µm) (Merck, Darmstadt, Germany), L-2350 thermostat (Merck Hitachi, Tokyo, Japan), L-7100 pump (Merck Hitachi, Tokyo, Japan), and VWR7614 degasser (Merck Hitachi, Tokyo, Japan) was used for the measurements. To quantify ergothioneine, a mobile phase consisting of two solvents—designated as A and B—was utilized. Solvent A comprised a water–to-methanol mixture (99:1, *v/v*) with 3.0 g of boric acid added to adjust the pH to 5.0. Solvent B consisted of methanol of HPLC grade. The gradient elution was programmed as follows: 100% A from 0 to 7 min, shifting to 70% A and 30% B from 7 to 10 min, then 10% A and 90% B from 10 to 14 min, followed by 100% B from 14 to 19 min. The gradient was then adjusted to 40% A and 60% B between 19 and 21 min, 70% A and 30% B from 21 to 26 min, and returned to 100% A from 26 to 45 min. The flow rate of the mobile phase was maintained at 0.5 mL/min, the column temperature was maintained at 25 °C and detection was performed at a wavelength of 257 nm.

#### 3.5.2. Analysis of Lovastatin

The analysis of lovastatin content was conducted using RP-HPLC method using a Merck Hitachi LaChrom liquid chromatography system equipped with a UV L-7400 detector, RP-18e 4 × 250 mm column (Purospher^®^, particle size 5 µm), L-2350 thermostat, L-7100 pump, and VWR7614 degasser, as described by Pansuriya and Singhal [27]. Extracts (methanolic, ethanolic, and dual extracts dissolved in various solvents) were analyzed. Isocratic elution was applied, using an eluent mixture of acetonitrile and 0.1% phosphoric acid solution in a 60:40 (*v/v*) ratio. The detection wavelength was set to λ = 238 nm, and the column temperature was maintained at 25 °C. The precise method for determining the content of lovastatin has been described previously [46].

#### 3.5.3. Analysis of Indole Compounds

For the analysis of L-tryptophan, isocratic elution was applied using a mixture of methanol, water, and 0.1 mol/L ammonium acetate in a volumetric ratio of 15:14:1 using a VWR/Hitachi LaChrom Elite liquid chromatography system equipped with a DAD L-2455 detector, a Purospher^®^ RP-18e (4 × 250 mm, particle size 5 µm) column, an L-2350 thermostat, an L-2130 pump, and an L-2200 autosampler. Process parameters included a wavelength of 280 nm, a sample injection volume of 20 µL, a column temperature of 25 °C, and a mobile phase flow rate of 1.0 mL/min. For the analysis of serotonin and 5-HTP, isocratic elution was used with an eluent composed of 0.1% phosphoric acid and acetonitrile in a volumetric ratio of 97:3 using a Merck Hitachi LaChrom liquid chromatography system equipped with a UV L-7400 detector, RP-18e 4 × 250 mm column (Purospher^®^, particle size 5 µm), L-2350 thermostat, L-7100 pump, and VWR7614 degasser. Parameters for this analysis included a wavelength of 275 nm, a sample injection volume of 20 µL, a column temperature of 25 °C, and a mobile phase flow rate of 1.0 mL/min. The precise method for determining the content of indole compounds has been described previously [46].

#### 3.5.4. Analysis of L-Phenylalanine

The analysis of L-phenylalanine was conducted using a VWR/Hitachi LaChrom Elite liquid chromatography system equipped with a DAD L-2455 detector, according to the method described by Ellnain-Wojtaszek and Zgórka [47]. A Purospher^®^ RP-18e (4 × 250 mm, particle size 5 µm) column, an L-2350 thermostat, an L-2130 pump, and an L-2200 autosampler were utilized. Gradient elution was applied using two eluents: methanol–0.5% acetic acid (1:4 *v/v*) as eluent A and methanol as eluent B. The wavelength was set to 254 nm, and the column temperature was maintained at 25 °C. The gradient program included the following stages: 0 min—100% A, 0% B; 20 min—100% A, 0% B; 35 min—80% A, 20% B; 45 min—70% A, 30% B; 55 min—60% A, 40% B; 60 min—50% A, 50% B; 65 min—25% A, 75% B; 70 min—0% A, 100% B; 75 min—0% A, 100% B; 80 min—100% A, 0% B; and 90 min—100% A, 0% B. The flow rate was set at 1 mL/min, and the injection volume was 20 µL. 

#### 3.5.5. Analysis of Sterols

The determinations of sterols were performed using a VWR/Hitachi LaChrom Elite liquid chromatography system equipped with a DAD L-2455 detector, a Purospher^®^ RP-18e (4 × 250 mm, particle size 5 µm) column, an L-2350 thermostat, an L-2130 pump, and an L-2200 autosampler. according to Yuan et al. [48]. Gradient elution was applied, using two solvents: solvent A, a mixture of methanol and water in an 8:2 (*v/v*) ratio, and solvent B, a mixture of methanol and dichloromethane in a 75:25 (*v/v*) ratio. The flow rate was 1 mL/min, the sample injection volume was 20 µL, the detection wavelength was λ = 280 nm, and the column temperature was maintained at 30 °C. The elution gradient program was as follows: initially, 100% of solvent A and 0% of solvent B were set and maintained for 10 min. Then, over the next 10 min, the proportions were gradually adjusted to 60% solvent A and 40% solvent B. In the subsequent stage, lasting 10 min, the proportions were changed to 40% solvent A and 60% solvent B, followed by an adjustment over the next 10 min to 20% solvent A and 80% solvent B. In the final phase, lasting 10 min, the proportions were changed to 0% solvent A and 100% solvent B, which was maintained for an additional 10 min. After completing the gradient program, the column was conditioned for 10 min under the initial conditions (100% solvent A and 0% solvent B).

### 3.6. Bioelement Analysis

To determine the content of selected bioelements—such as potassium (K), calcium (Ca), titanium (Ti), zinc (Zn), rubidium (Rb), iron (Fe), copper (Cu), nickel (Ni), strontium (Sr), selenium (Se), manganese (Mn), bromine (Br), and chromium (Cr)—the analyzed samples were subjected to mineralization. To prepare samples for mineralization, exactly 0.2 g of each sample was weighed, with the process conducted in three replicates. In the conducted experiment, the wet mineralization method was employed. A Magnum II microwave apparatus (ERTEC-Poland, Wrocław, Poland) was utilized for this process. The mineralization process was conducted at a temperature of 290 °C and consisted of three 10-minute stages. Upon completion, the resulting solutions were transferred to quartz evaporators and evaporated at 150 °C using a hot plate. Volumetric flasks with a capacity of 10 mL were prepared, and the residue obtained after the mineralization process was quantitatively transferred into them. The flasks were then filled with water that had undergone a quadruple distillation process. The elemental composition was determined using a total reflection X-ray fluorescence (TXRF) Nanohunter II spectrometer (Rigaku, Wrocław, Poland), equipped with an X-ray lamp featuring a molybdenum anode. Measurements were conducted for 1000 s at a voltage of 50 kV.

### 3.7. Analysis of β-Glucans

The β-glucan content was determined using a test kit (Megazyme© Ltd., Bray, County Wicklow, Ireland) according to the manufacturer’s instructions and the procedure described by Sari et al. [4]. A 0.1 g portion of previously lyophilized mushroom material or dual extract (in paste form) was weighted. Subsequently, 1.5 mL of 37% HCl was added, and the mixture was heated at 30 °C for 45 min. In the next step, 10 mL of distilled water was added to each sample, and the mixture was incubated for 2 h in a boiling water bath. After neutralization with 2 M KOH, acetate buffer (pH = 5) was added to the *H. erinaceus* samples to obtain a final volume. Next, a 0.1 mL aliquot of the solution was taken and mixed with exo-1,3-β-glucanase (20 U/mL) and β-glucosidase (20 U/mL). The solution was then incubated in a water bath at 40 °C for 1 h. To each analyzed sample, 3 mL of Megazyme glucose determination reagent (glucose oxidase/peroxidase; GOPOD) was added, and the mixture was further incubated at 50 °C for 20 min. Following the addition of 8 mL of acetate buffer (pH = 3.8) and 0.2 mL of amyloglucosidase (1630 U/mL), the samples were incubated in a water bath at 50 °C for 30 min. In the next step, a 0.1 mL aliquot was taken and mixed with 0.1 mL of acetate buffer (pH = 5.0) and 3 mL of GOPOD. The resulting solution was incubated again at 50 °C for 20 min. The samples were analyzed using a UV/VIS spectrophotometer A560 (AOE Instruments Co., Ltd., Shanghai, China) at a wavelength of λ = 510 nm and compared with a blank sample. The β-glucan content was calculated according to the manufacturer’s instructions. The enzymatic test method for detecting [(1-3)(1-4)]-β-D-glucans was applied, which is an effective method for quantitatively determining β-glucans in mushrooms, with a standard error of the method being <5% (Megazyme© International Ireland Ltd., 2013; Wicklow, Ireland).

### 3.8. Antioxidant Analysis 

#### 3.8.1. Determination of Antioxidant Potential Using the DPPH Method 

The determination of antioxidant activity was performed according to the method by Molyneux, utilizing the DPPH radical (2,2-diphenyl-1-picrylhydrazyl) from Sigma-Aldrich (St. Louis, MO, USA) [49]. In this method, 0.1 mL of the prepared fungal supernatant was dissolved in an appropriate amount of solvent and thoroughly mixed (with experimental adjustments to dilutions). Then, 0.1 mL of the resulting solution was mixed with 4.9 mL of a DPPH^•^ solution at a concentration of 0.1 mM. The reaction mixture was shaken and incubated in the dark at room temperature for 30 min. The absorbance of the reaction mixture was measured at a wavelength of 517 nm against a control sample, using a UV/Vis spectrophotometer A560 (AOE Instrument, Shanghai, China). Antioxidant activity was calculated using the following equation: DPPH [%] = [(A0 − A1)/A0] × 100, where A0 represents the absorbance of the reference solution, and A1 represents the absorbance of the test solution. Each sample was tested in triplicate to ensure reproducibility. The total antioxidant activity was expressed as ascorbic acid equivalents (mg AAE/g dry weight), based on a calibration curve constructed using standard solutions of ascorbic acid in the concentration range of 5–25 µg/mL.

#### 3.8.2. Determination of Antioxidant Potential Using the ABTS Method 

The determination of antioxidant activity using the ABTS method was conducted following the modified method of Thaipong et al. [50]. Stock solutions of 7.4 mM ABTS from Sigma-Aldrich (St. Louis, MO, USA) and 2.6 mM potassium persulfate were prepared. These solutions were mixed in equal volumetric proportions and allowed to react for 24 h in darkness at room temperature. The resulting solution was then diluted with methanol to achieve an absorbance of 1.1 units at a wavelength of 734 nm, measured using a UV/Vis spectrophotometer A560 (AOE Instrument, Shanghai, China). Fresh ABTS^•+^ solution was prepared for each assay. Fungal extracts were diluted with methanol, and 0.1 mL of the diluted extracts was reacted with 2.9 mL of the previously prepared ABTS^•+^ solution for 2 h in darkness. Absorbance was measured at a wavelength of 734 nm using a UV/Vis spectrophotometer A560 (AOE Instrument, Shanghai, China). Each sample was tested in triplicate to ensure reproducibility. The total antioxidant activity was expressed as ascorbic acid equivalents (mg AAE/g dry weight), based on a calibration curve constructed using standard solutions of ascorbic acid in the concentration range of 10–100 µg/mL.

### 3.9. Analysis of Total Phenolic Compound Content

The Folin–Ciocalteu method was employed to determine the total phenolic content [51]. To perform the analysis, 0.1 mL of the prepared extract was mixed with 2 mL of sodium carbonate, followed by the addition of Folin–Ciocalteu reagent (0.1 mL) mixed with deionized water in a 1:1 ratio (*v/v*) to the test tubes. The reaction mixture was then incubated at room temperature in the dark for 45 min. After the incubation period, the absorbance of the samples was measured at 750 nm using a Helios-β spectrophotometer (Thermofisher, Altrincham, UK). Each sample was analyzed in triplicate to ensure reproducibility. The total phenolic content was quantified using a calibration curve prepared with standard solutions of gallic acid in the concentration range of 100–500 µg/mL, and the results were expressed as gallic acid equivalents (mg GAE/g dry weight) of *H. erinaceus* samples.

### 3.10. Statistical Analysis 

The samples were analyzed in three independent replicates. Statistical analysis of the obtained results was performed using two-way analysis of variance (ANOVA), where the dependent variable was the compound content and the independent variables were the type of cultivation substrate and the method of sample preparation. The significance level was set at *p* < 0.05. The analyses were carried out using STATISTICA v. 14 (StatSoft Inc., Tulsa, OK, USA).

## 4. Conclusions

Dual extraction using moderate ethanol concentrations proved most effective for isolating key bioactive compounds from *H. erinaceus*, including ergothioneine, lovastatin, L-phenylalanine, and ergosterol, while no significant effect of ethanol concentration was observed on antioxidant activity during the extraction process. While dual extracts showed enhanced levels of certain antioxidants and bioactive metabolites, whole fruiting bodies contained higher amounts of essential bioelements. When selecting substrates, targeted effects should be considered; for example, to obtain high amounts of ergothioneine, hemp straw should be used as the primary substrate component, whereas beech sawdust is preferable for maximizing ergosterol content. Considering all obtained results, it can be concluded that most of the analyzed compounds occur in greater amounts in fruiting bodies cultivated on beech sawdust compared to those grown on hemp straw. When comparing the bioelement content in *H. erinaceus* fruiting bodies, higher concentrations of zinc, rubidium, iron, and copper were observed in specimens cultivated on a substrate containing hemp straw. When comparing the extraction methods, the double extraction procedure yielded higher concentrations of all analyzed elements, with the exception of nickel. These findings confirm that both cultivation substrate and extraction method significantly influence the bioactive profile of *H. erinaceus*, highlighting its potential as a valuable source of health-promoting compounds. The obtained results undoubtedly confirm the feasibility of utilizing post-production by-products. This approach contributes to the maintenance of a sustainable waste management system, thereby supporting environmental protection, and—importantly—does not negatively affect the content of bioactive compounds in the cultivated mushrooms. Such experimental results enable companies involved in the production of natural raw materials to reduce production costs and facilitate more efficient waste management. The results obtained are satisfactory and demonstrate the potential for utilizing industrial by-products.

## Figures and Tables

**Figure 1:**
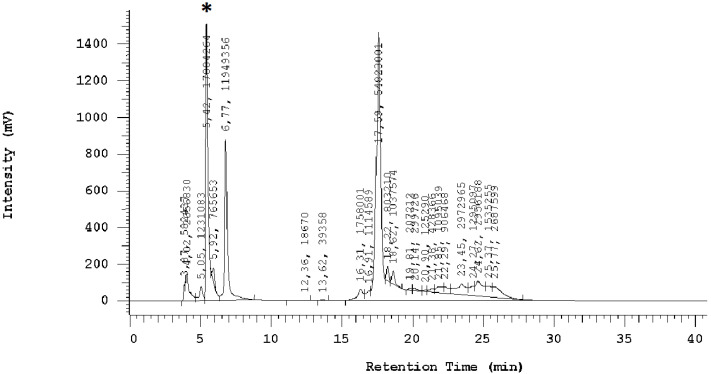
HPLC chromatogram of ergothioneine obtained from *H. erinaceus* fruiting bodies grown on beech sawdust, extracted with 95% ethanol as part of dual extract preparation (*—peak recorded for ergothioneine).

**Table 1:** The content of bioactive compounds in *Hericium erinaceus* extracts obtained from fruiting bodies cultivated on substrates supplemented with hemp straw and beech sawdust was analyzed in dual water–ethanol extracts (prepared by dissolution in either 96% ethanol or a 70:30 ethanol–water mixture), as well as in traditionally prepared liquid methanolic and ethanolic extracts [expressed in mg per 100 g of dry fruiting bodies or per 100 g of dual extract].

	HeDeHS70E	HeDeHS80E	HeDeHS95E	HeDeBS70E	HeDeBS80E	HeDeBS95E	HeDeHS70EW	HeDeHS80EW	HeDeHS95EW	HeDeBS70EW	HeDeBS80EW	HeDeBS95EW	HeMeHS	HeMeBS	HeEeHS	HeEeBS
Ergothioneine	583 ± 3 ^g^	715 ± 5 ^f^	326 ± 1 ^h^	259 ± 1 ^i^	233 ± 1 ^i^	261 ± 4 ^i^	1558 ± 19 ^a^	1388 ± 29 ^b^	1200 ± 4 ^c^	1397 ± 2 ^b^	1163 ± 3 ^c^	984 ± 8 ^d^	861 ± 65 ^e^	853 ± 34 ^e^	250 ± 23 ^i^	150 ± 4 ^j^
Lovastatin	3.12 ± 0.05 ^a^	2.49 ± 0.01 ^b^	3.21 ± 0.01 ^a^	3.23 ± 0.02 ^a^	3.15 ± 0.06 ^a^	3.27 ± 0.03 ^a^	2.74 ± 0.01 ^b^	2.70 ± 0.02 ^b^	3.08 ± 0.24 ^a^	3.05 ± 0.21 ^a^	3.23 ± 0.05 ^a^	3.23 ± 0.02 ^a^	2.23 ± 0.03 ^c^	1.24 ± 0.02 ^d^	2.20 ± 0.03 ^c^	1.11 ± 0.04 ^d^
L-Tryptophan	*	*	*	*	*	*	–	–	*	–	–	1.54 ± 0.23 ^c^	8.36 ± 1.2 ^b^	17.0 ± 0.1 ^a^	1.25 ± 0.08 ^c^	2.42 ± 0.29 ^c^
5-Hydroxy-L-tryptophan	–	–	–	–	–	–	–	*	–	–	–	*	*	*	–	–
Serotonin	–	–	11.78 ± 0.57 ^d^	–	–	–	–	26.3 ± 0.3 ^b^	–	31.6 ± 0.2 ^a^	–	–	–	13.9 ± 0.1 ^c^	–	–
L-Phenylalanine	353 ± 25 ^d^	161 ± 1 ^h^	422 ± 7 ^c^	460 ± 14 ^b^	23.6 ± 0.2 ^j^	531 ± 29 ^a^	85.7 ± 1.4 ^i^	29.0 ± 0.8 ^j^	422 ± 7 ^c^	84.7 ± 0.4 ^i^	288 ± 12 ^f^	325 ± 1 ^d, e^	296 ± 0 ^e, f^	311 ± 10 ^e, f^	154 ± 2 ^h^	234 ± 2 ^g^

*n* = 3; —mean not detected; * —trace amounts; **HeDeHS70**—dual extract dissolved in (**E**) HPLC-grade 96% ethanol or (**EW**) 70:30 ethanol–water mixture (dual extract originally obtained using a 70% ethanol solution) prepared from *Hericium erinaceus* fruiting bodies cultivated on a hemp straw substrate; **HeDeHS80**—dual extract dissolved in (**E**) HPLC-grade 96% ethanol or (**EW**) 70:30 ethanol–water mixture (dual extract originally obtained using a 80% ethanol solution) prepared from *Hericium erinaceus* fruiting bodies cultivated on a hemp straw substrate; **HeDeHS95**—dual extract dissolved in (**E**) HPLC-grade 96% ethanol or (**EW**) 70:30 ethanol–water mixture (dual extract originally obtained using a 95% ethanol solution) prepared from *Hericium erinaceus* fruiting bodies cultivated on a hemp straw substrate; **HeDeBS70**—dual extract dissolved in (**E**) HPLC-grade 96% ethanol or (**EW**) 70:30 ethanol–water mixture (dual extract originally obtained using a 70% ethanol solution) prepared from *Hericium erinaceus* fruiting bodies cultivated on a beech sawdust substrate; **HeDeBS80**—dual extract dissolved in (**E**) HPLC-grade 96% ethanol or (**EW**) 70:30 ethanol–water mixture (dual extract originally obtained using a 80% ethanol solution) prepared from *Hericium erinaceus* fruiting bodies cultivated on a beech sawdust substrate; **HeDeBS95**—dual extract dissolved in (**E**) HPLC-grade 96% ethanol or (**EW**) 70:30 ethanol–water mixture (dual extract originally obtained using a 95% ethanol solution) prepared from *Hericium erinaceus* fruiting bodies cultivated on a beech sawdust substrate; **HeMeHS**—liquid methanolic extract obtained from *Hericium erinaceus* fruiting bodies cultivated on a hemp straw substrate; **HeMeBS**—liquid methanolic extract obtained from *Hericium erinaceus* fruiting bodies cultivated on a beech sawdust substrate; **HeEeHS**—liquid ethanolic extract obtained from *Hericium erinaceus* fruiting bodies cultivated on a hemp straw substrate; **HeEeBS**—liquid ethanolic extract obtained from *Hericium erinaceus* fruiting bodies cultivated on a beech sawdust substrate; the different letters next to the values represent Tukey’s HSD post hoc results (*p* < 0.05) and indicate statistically significant differences between means within the same row.

**Table 3:** Determination of total phenolic content, antioxidant activity, and β-glucan levels in *Hericium erinaceus* extracts obtained from fruiting bodies cultivated on substrates supplemented with hemp straw and beech sawdust, analyzed in dual water–ethanol extracts (dissolved in either 96% ethanol or 70:30 ethanol–water mixture) and traditionally prepared liquid methanolic and ethanolic extracts.

HeDeHS70E	HeDeHS80E	HeDeHS95E	HeDeBS70E	HeDeBS80E	HeDeBS95E	HeDeHS70EW	HeDeHS80EW	HeDeHS95EW	HeDeBS70EW	HeDeBS80EW	HeDeBS95EW	HeMeHS	HeMeBS	HeEeHS	HeEeBS
Total phenolic compound content—gallic acid equivalent (GAE) mg/g of dry weight															
25.4 ± 0.5 ^g, h^	38.7 ± 0.3 ^f^	24.0 ± 0.5 ^g, h^	26.1 ± 0.7 ^g^	17.4 ± 0.2 ^i^	22.2 ± 0.7 ^h^	81.3 ± 1.6 ^b^	73.5 ± 1.6 ^c^	79.7 ± 0.2 ^b^	89.1 ± 0.3 ^a^	80.4 ± 1.1 ^b^	87.2 ± 0.6 ^a^	42.7 ± 0.3 ^e^	68.1 ± 3.1 ^d^	13.6 ± 0.1 ^j^	11.7 ± 0.5 ^j^
DPPH antioxidant activity—ascorbic acid equivalent (AAE) mg/g of dry weight															
5.23 ± 0.21 ^d, e^	4.40 ± 0.04 ^f^	5.58 ± 0.17 ^d^	5.44 ± 0.15 ^d, e^	4.98 ± 0.20 ^e^	5.44 ± 0.19 ^d, e^	7.14 ± 0.29 ^b, c^	6.75 ± 0.33 ^c^	7.09 ± 0.33 ^b, c^	8.03 ± 0.20 ^a^	7.35 ± 0.14 ^b^	7.08 ± 0.16 ^b, c^	2.02 ± 0.08 ^g^	2.07 ± 0.07 ^g^	1.42 ± 0.03 ^h^	1.24 ± 0.09 ^h^
ABTS antioxidant activity—ascorbic acid equivalent (AAE) mg/g of dry weight															
2.31 ± 0.01 ^g^	3.07 ± 0.24 ^f, g^	2.03 ± 0.08 ^g^	2.25 ± 0.06 ^g^	1.73 ± 0.06 ^g^	1.96 ± 0.10 ^g^	23.1 ± 0.6 ^a^	18.0 ± 0.7 ^c^	20.6 ± 1.5 ^b^	23.5 ± 1.4 ^a^	23.8 ± 0.9 ^a^	23.5 ± 0.5 ^a^	5.53 ± 0.37 ^e^	10.7 ± 0.7 ^d^	4.95 ± 0.25 ^e, f^	2.57 ± 0.05 ^g^
[(1-3)(1-4)]-β-D-glucans expressed as g/100 g of dual extract/dried fruiting bodies															
**HeDeHS70**		**HeDeHS80**		**HeDeHS95**		**HeDsBS70**		**HeDsBS80**		**HeDsBS95**		**HeFbHS**		**HeFbBS**	
0.186 ± 0.009 ^c, d^		0.160 ± 0.023 ^d^		0.391 ± 0.038 ^a^		0.262 ± 0.081 ^b, c^		0.117 ± 0.043 ^d^		0.323 ± 0.006 ^a, b^		0.131 ± 0.035 ^d^		0.129 ± 0.009 ^d^	

*n* = 3; detailed description can be found below Table 1 and Table 2; the different letters next to the values represent Tukey’s HSD post hoc results (*p* < 0.05) and indicate statistically significant differences between means within the same row.

## Data Availability

The original contributions presented in this study are included in the article. Further inquiries can be directed to the corresponding authors.

## Supplementary Materials

The following supporting information can be downloaded at https://www.mdpi.com/article/10.3390/molecules30153094/s1, Figure S1: Chromatogram of the separation of ergothioneine for HeDeBS70E. Figure S2: Chromatogram of the separation of ergothioneine for HeDeBS80E. Figure S3: Chromatogram of the separation of ergothioneine for HeDeBS95E. Figure S4: Chromatogram of the separation of ergothioneine for HeDeHS95E. Figure S5: Chromatogram of the separation of ergothioneine for HeMeBS. Figure S6: Chromatogram of the separation of ergothioneine for HeDeBS80E. Figure S7: Chromatogram of the separation of ergothioneine for HeDeHS80E. Figure S8: Chromatogram of the separation of ergothioneine for HeDeHS70EW. Figure S9: Chromatogram of the separation of ergothioneine for HeDeHS80EW. Figure S10: Chromatogram of the separation of ergothioneine for HeDeHS95EW. Figure S11: Chromatogram of the separation of ergothioneine for HeDeBS70EW. Figure S12: Chromatogram of the separation of ergothioneine for HeDeBS80EW. Figure S13: Chromatogram of the separation of ergothioneine for HeDeBS95EW. Figure S14: Chromatogram of the separation of ergothioneine for HeMeHS. Figure S15: Chromatogram of the separation of ergothioneine for HeEeHS. Figure S16: Chromatogram of the separation of ergothioneine for HeEeBs. Figure S17: Chromatogram of the separation of lovastatin for HeDeHS70E. Figure S18: Chromatogram of the separation of lovastatin for HeDeHS80E. Figure S19: Chromatogram of the separation of lovastatin for HeDeHS95E. Figure S20: Chromatogram of the separation of lovastatin for HeDeBS80E. Figure S21: Chromatogram of the separation of lovastatin for HeDeBS70E. Figure S22: Chromatogram of the separation of lovastatin for HeDeBS95E. Figure S23: Chromatogram of the separation of lovastatin for HeDeHS70EW. Figure S24: Chromatogram of the separation of lovastatin for HeDeHS80EW. Figure S25: Chromatogram of the separation of lovastatin for HeDeHS95EW. Figure S26: Chromatogram of the separation of lovastatin for HeDeBS70EW. Figure S27: Chromatogram of the separation of lovastatin for HeDeBS80EW. Figure S28: Chromatogram of the separation of lovastatin for HeDeBS95EW. Figure S29: Chromatogram of the separation of lovastatin for HeMeHS. Figure S30: Chromatogram of the separation of lovastatin for HeMeBS. Figure S31: Chromatogram of the separation of lovastatin for HeEeHs. Figure S32: Chromatogram of the separation of lovastatin for HeEeBs. Figure S33: Chromatogram of the separation of serotonin for HeDeBS80EW. Figure S34: Chromatogram of the separation of serotonin for HeDeHS80EW. Figure S35: Chromatogram of the separation of serotonin for HeMeBS. Figure S36: Chromatogram of the separation of serotonin for HeDeHS95E. Figure S37: Chromatogram of the separation of L-phenylalanine for HeDeHS70E. Figure S38: Chromatogram of the separation of L-phenylalanine for HeDeHS80E. Figure S39: Chromatogram of the separation of L-phenylalanine for HeDeHS95E. Figure S40: Chromatogram of the separation of L-phenylalanine for HeDeBS70E. Figure S41: Chromatogram of the separation of L-phenylalanine for HeDeBS80E. Figure S42: Chromatogram of the separation of L-phenylalanine for HeDeBS95E. Figure S43: Chromatogram of the separation of L-phenylalanine for HeDeHS70EW. Figure S44: Chromatogram of the separation of L-phenylalanine for HeDeHS80EW. Figure S45: Chromatogram of the separation of L-phenylalanine for HeDeHS95EW. Figure S46: Chromatogram of the separation of L-phenylalanine for HeDeBS70EW. Figure S47: Chromatogram of the separation of L-phenylalanine for HeDeBS80EW. Figure S48: Chromatogram of the separation of L-phenylalanine for HeDeBS95EW. Figure S49: Chromatogram of the separation of L-phenylalanine for HeMeHS. Figure S50: Chromatogram of the separation of L-phenylalanine for HeMeBS. Figure S51: Chromatogram of the separation of L-phenylalanine for HeEeHS. Figure S52: Chromatogram of the separation of L-phenylalanine for HeEeBS. Figure S53: Chromatogram of the separation of tocopherol (*) and ergosterol (**) for HeDeHS70E. Figure S54: Chromatogram of the separation of tocopherol (*) and ergosterol (**) for HeDeHS80E. Figure S55: Chromatogram of the separation of tocopherol (*) and ergosterol (**) for HeDeHS95E. Figure S56: Chromatogram of the separation of tocopherol (*) and ergosterol (**) for HeDeBS70E. Figure S57: Chromatogram of the separation of tocopherol (*) and ergosterol (**) for HeDeBS70E. Figure S58: Chromatogram of the separation of tocopherol (*) and ergosterol (**) for HeDeBS70E. Figure S59: Chromatogram of the separation of tocopherol (*) and ergosterol (**) for HEFbHS. Figure S60: Chromatogram of the separation of tocopherol (*) and ergosterol (**) for HEFbBS.

## Abbreviations

The following abbreviations are used in this manuscript:

5-HTP	5-Hydroxy-L-tryptophan
AAE	Ascorbic acid equivalent
ABTS	2,2-Azinobis (3-ethylbenzothiazoline-6-sulfonic acid)
ABTS^•+^	2,2-Azinobis (3-ethylbenzothiazoline-6-sulfonic acid) radical cation
AUC	Area under the curve
D.W.	Dry weight
DPPH^•^	2,2-Diphenyl-1-picrylhydrazyl radical
FDA	Food and Drug Administration
GAE	Gallic acid equivalent
HAT	Hydrogen atom transfer
LDL	Low-density lipoproteins
ROS	Reactive oxygen species
RP-HPLC	Reverse-phase high performance liquid chromatography
SET	Single-electron transfer
TXRF	Total reflection X-Ray fluorescence
