# Supplementary material for: Influence of Plant-Based Substrate Composition and Extraction Method on Accumulation of Bioactive Compounds in Hericium erinaceus (Bull.) Pers. Fruiting Bodies
Source: Molecules. 2025 Jul 24;30(15):3094. doi: 10.3390/molecules30153094 (PMC12348041; doi:10.3390/molecules30153094)
Supplement: Supplementary file 1 [file molecules-30-03094-s001.zip › molecules-3733132-supplementary.pdf]

SUPPLEMENTARY DATA

Influence of Plant-Based Substrate Composition and Extraction Method on Accumulation of Bioactive Compounds in *Hericium erinaceus* (Bull.) Pers. Fruiting Bodies

The chromatograms obtained as a result of the analyses are presented below. The following abbreviations were used in the description of the chromatograms:

- **HeDeHS70E** – dual extract dissolved in HPLC-grade 96% ethanol (dual extract originally obtained using a 70% ethanol solution) prepared from *Hericium erinaceus* fruiting bodies cultivated on a hemp straw substrate;
- **HeDeHS70EW** – dual extract dissolved in HPLC-grade 70:30 ethanol-water mixture (dual extract originally obtained using a 70% ethanol solution) prepared from *Hericium erinaceus* fruiting bodies cultivated on a hemp straw substrate;
- **HeDeHS80E** – dual extract dissolved in HPLC-grade 96% ethanol (dual extract originally obtained using a 80% ethanol solution) prepared from *Hericium erinaceus* fruiting bodies cultivated on a hemp straw substrate;
- **HeDeHS80EW** – dual extract dissolved in HPLC-grade 70:30 ethanol-water mixture (dual extract originally obtained using a 80% ethanol solution) prepared from *Hericium erinaceus* fruiting bodies cultivated on a hemp straw substrate;
- **HeDeHS95E** – dual extract dissolved in HPLC-grade 96% ethanol mixture (dual extract originally obtained using a 95% ethanol solution) prepared from *Hericium erinaceus* fruiting bodies cultivated on a hemp straw substrate;
- **HeDeHS95EW** – dual extract dissolved in 70:30 ethanol-water mixture (dual extract originally obtained using a 95% ethanol solution) prepared from *Hericium erinaceus* fruiting bodies cultivated on a hemp straw substrate;

- **HeDeBS70E** – dual extract dissolved in HPLC-grade 96% ethanol dual extract originally obtained using a 70% ethanol solution) prepared from *Hericium erinaceus* fruiting bodies cultivated on a beech sawdust substrate;
- **HeDeBS70EW** – dual extract dissolved in 70:30 ethanol-water mixture (dual extract originally obtained using a 70% ethanol solution) prepared from *Hericium erinaceus* fruiting bodies cultivated on a beech sawdust substrate;
- **HeDeBS80E** – dual extract dissolved in HPLC-grade 96% ethanol (dual extract originally obtained using a 80% ethanol solution) prepared from *Hericium erinaceus* fruiting bodies cultivated on a beech sawdust substrate;
- **HeDeBS80EW** – dual extract dissolved in 70:30 ethanol-water mixture (dual extract originally obtained using a 80% ethanol solution) prepared from *Hericium erinaceus* fruiting bodies cultivated on a beech sawdust substrate;
- **HeDeBS95E** – dual extract dissolved in HPLC-grade 96% ethanol (dual extract originally obtained using a 95% ethanol solution) prepared from *Hericium erinaceus* fruiting bodies cultivated on a beech sawdust substrate;
- **HeDeBS95EW** – dual extract dissolved in 70:30 ethanol-water mixture (dual extract originally obtained using a 95% ethanol solution) prepared from *Hericium erinaceus* fruiting bodies cultivated on a beech sawdust substrate;
- **HeMeHS** – liquid methanolic extract obtained from *Hericium erinaceus* fruiting bodies cultivated on a hemp straw substrate;
- **HeMeBS** – liquid methanolic extract obtained from *Hericium erinaceus* fruiting bodies cultivated on a beech sawdust substrate;
- **HeEeHS** – liquid ethanolic extract obtained from *Hericium erinaceus* fruiting bodies cultivated on a hemp straw substrate;
- **HeEeBS** – liquid ethanolic extract obtained from *Hericium erinaceus* fruiting bodies cultivated on a beech sawdust substrate;

peak recorder marked *

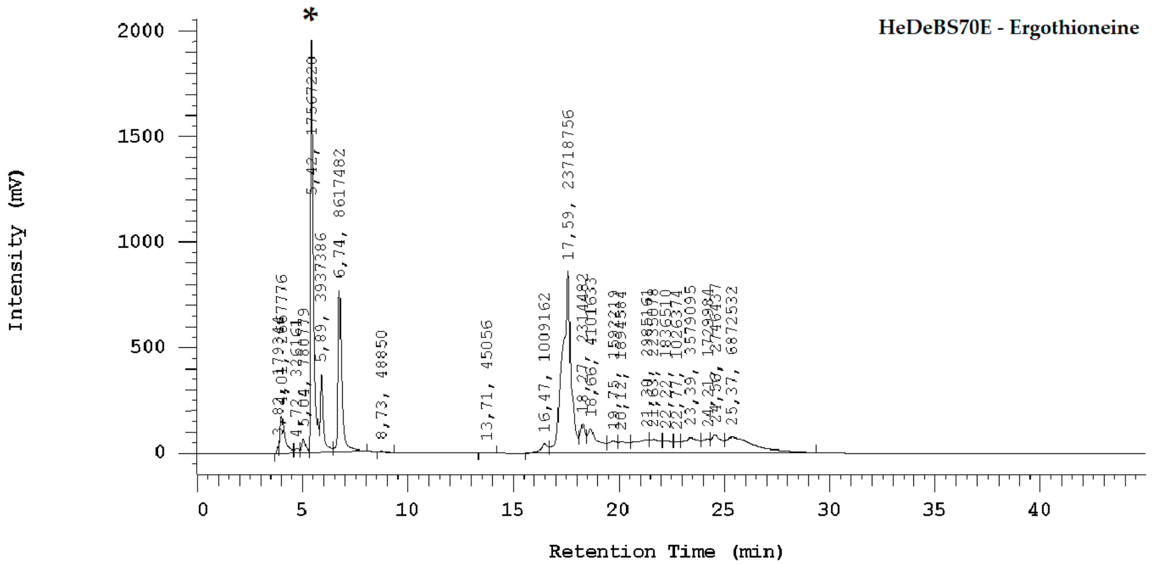

Figure S1. Chromatogram of the separation of ergothioneine for HeDeBS70E.

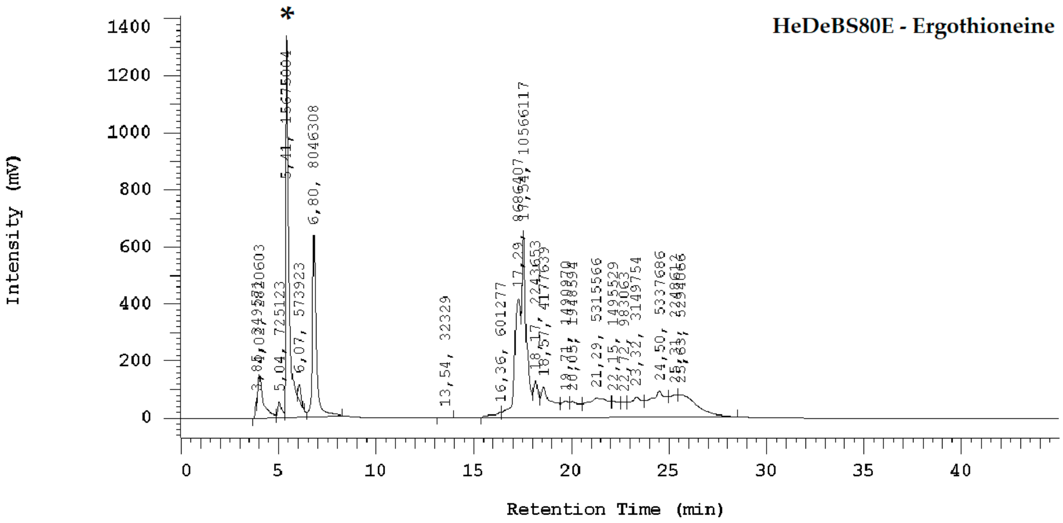

Figure S2. Chromatogram of the separation of ergothioneine for HeDeBS80E.

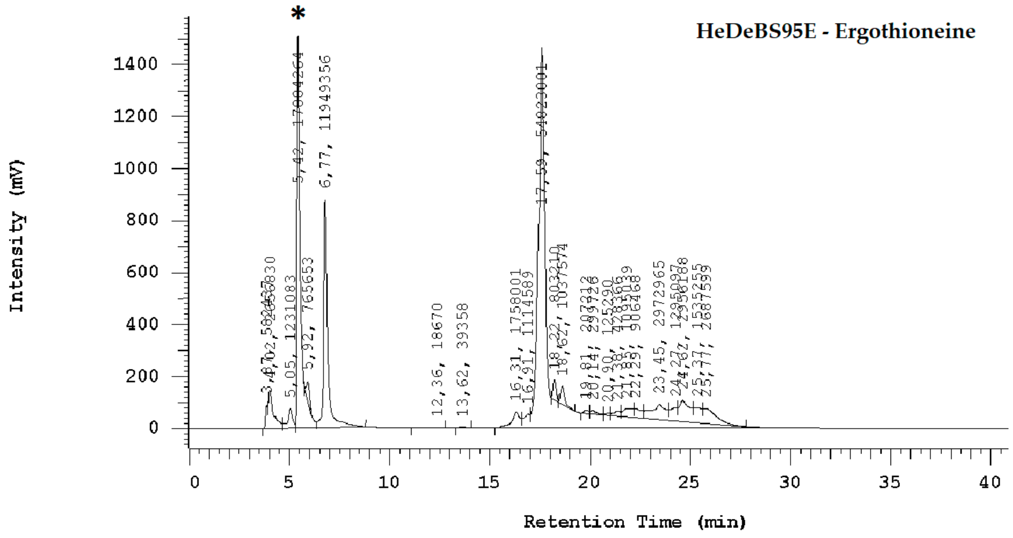

Figure S3. Chromatogram of the separation of ergothioneine for HeDeBS95E.

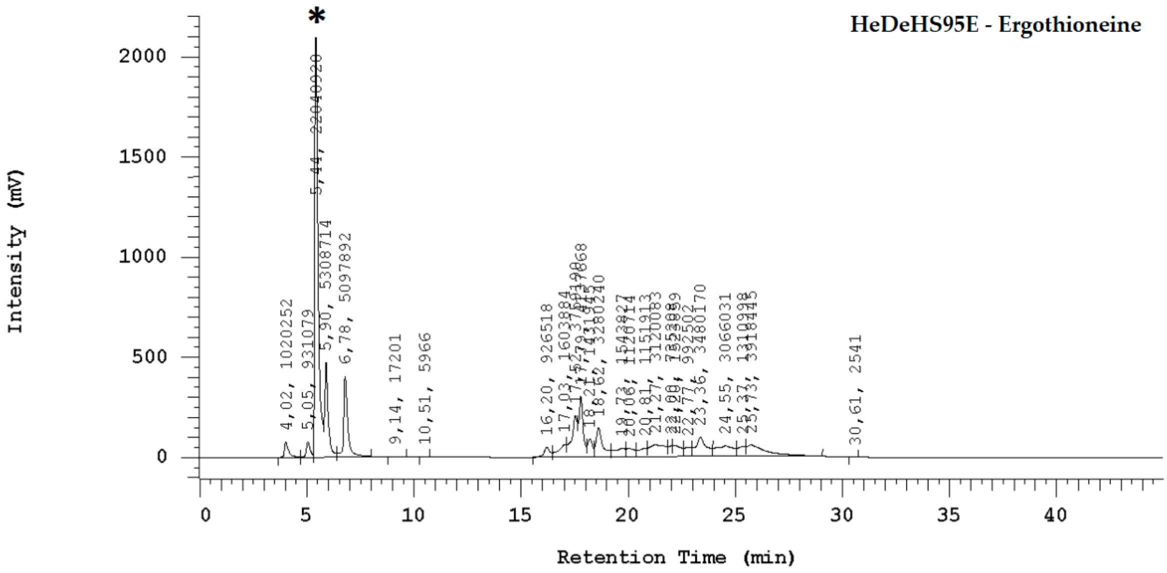

Figure S4. Chromatogram of the separation of ergothioneine for HeDeHS95E.

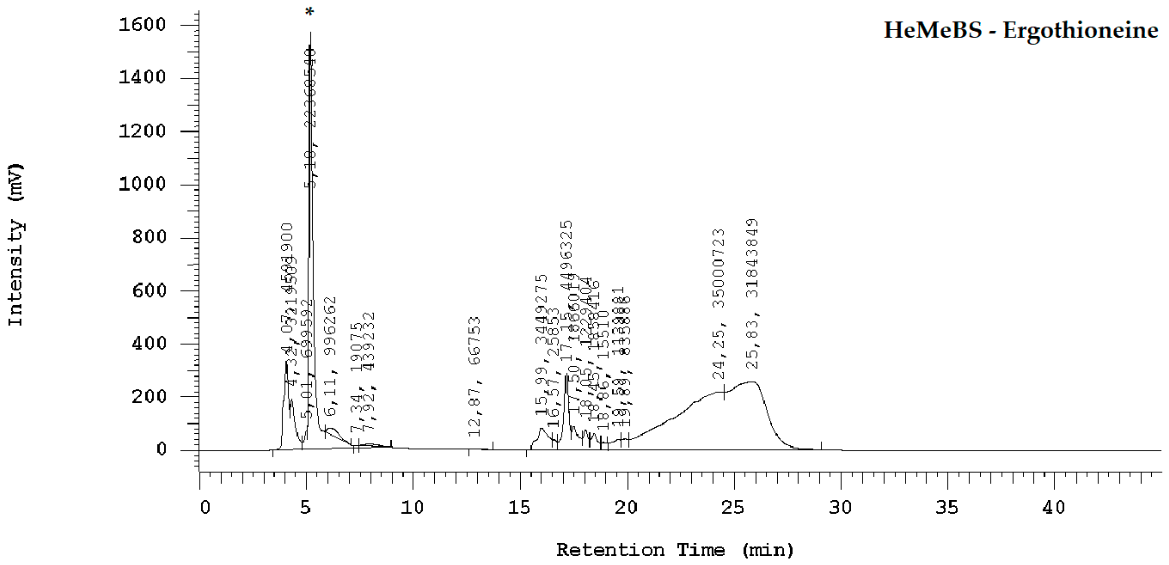

Figure S5. Chromatogram of the separation of ergothioneine for HeMeBS.

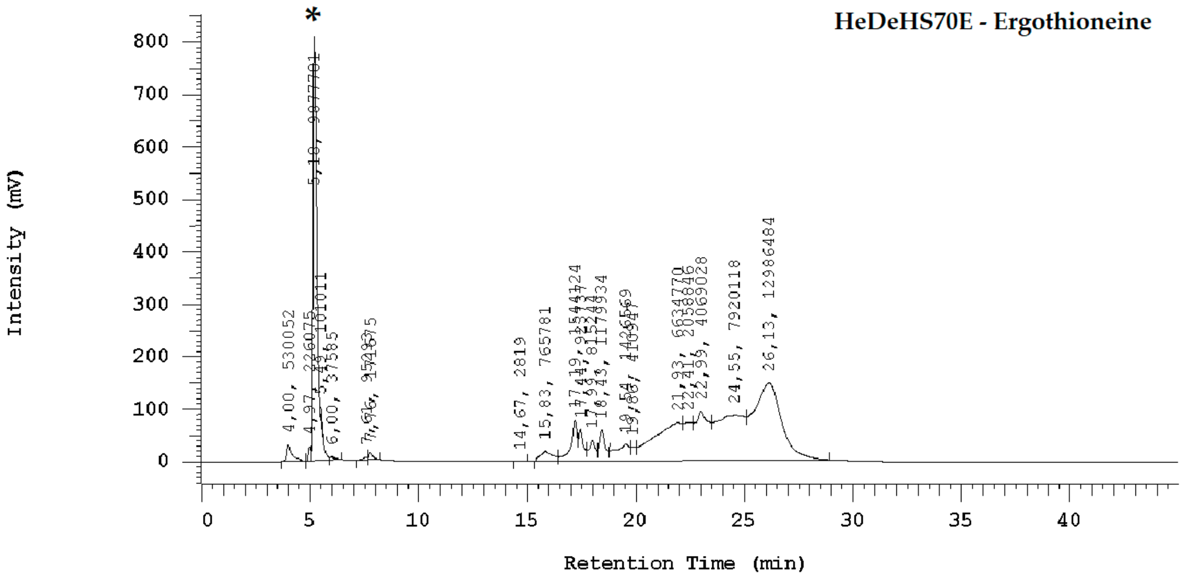

Figure S6. Chromatogram of the separation of ergothioneine for HeDeBS80E.

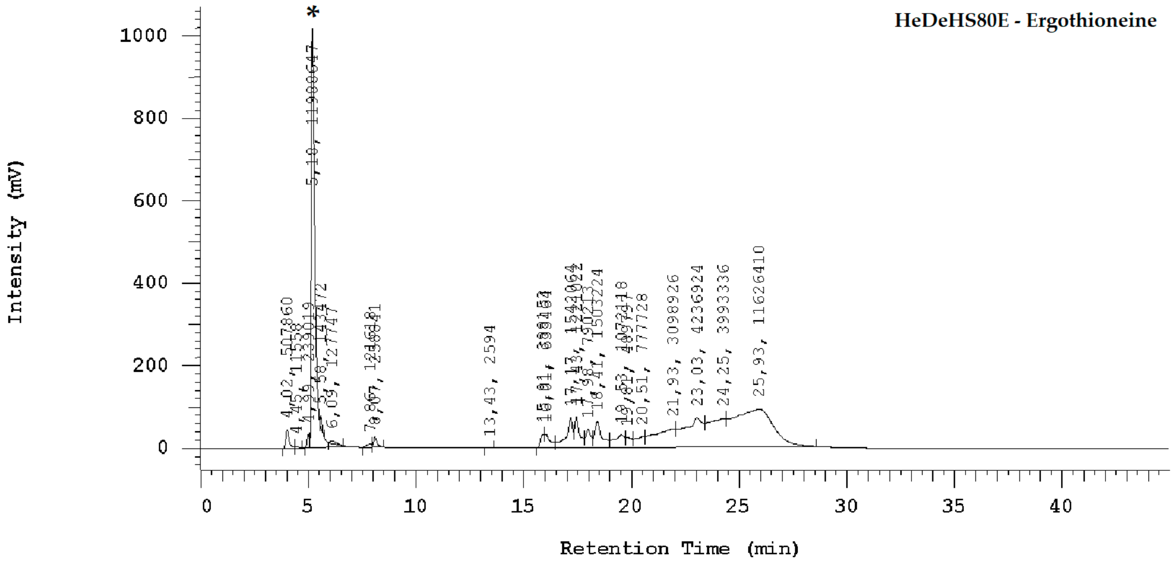

Figure S7. Chromatogram of the separation of ergothioneine for HeDeHS80E.

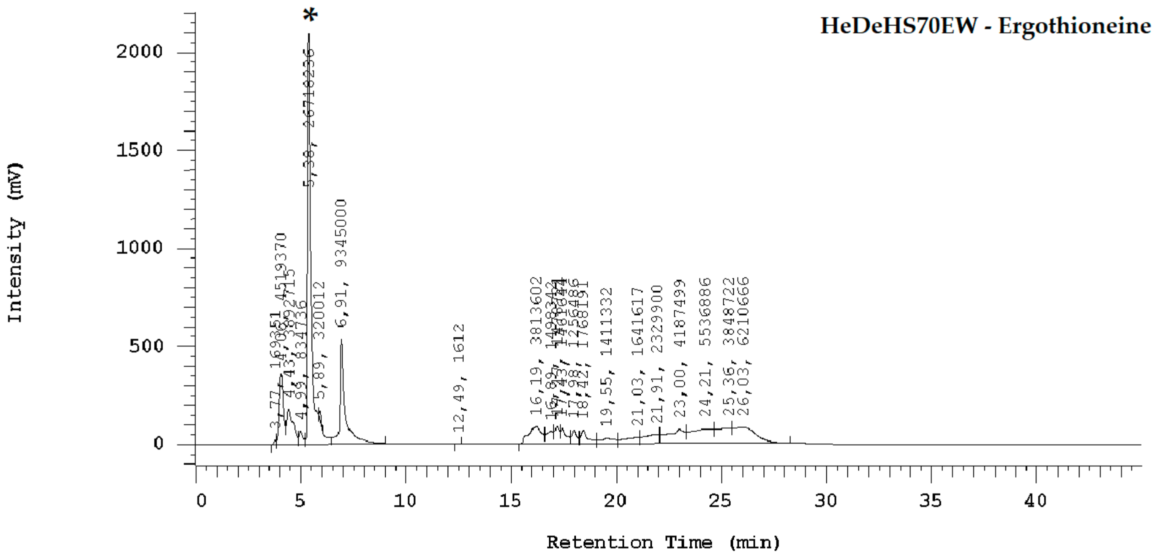

Figure S8. Chromatogram of the separation of ergothioneine for HeDeHS70EW.

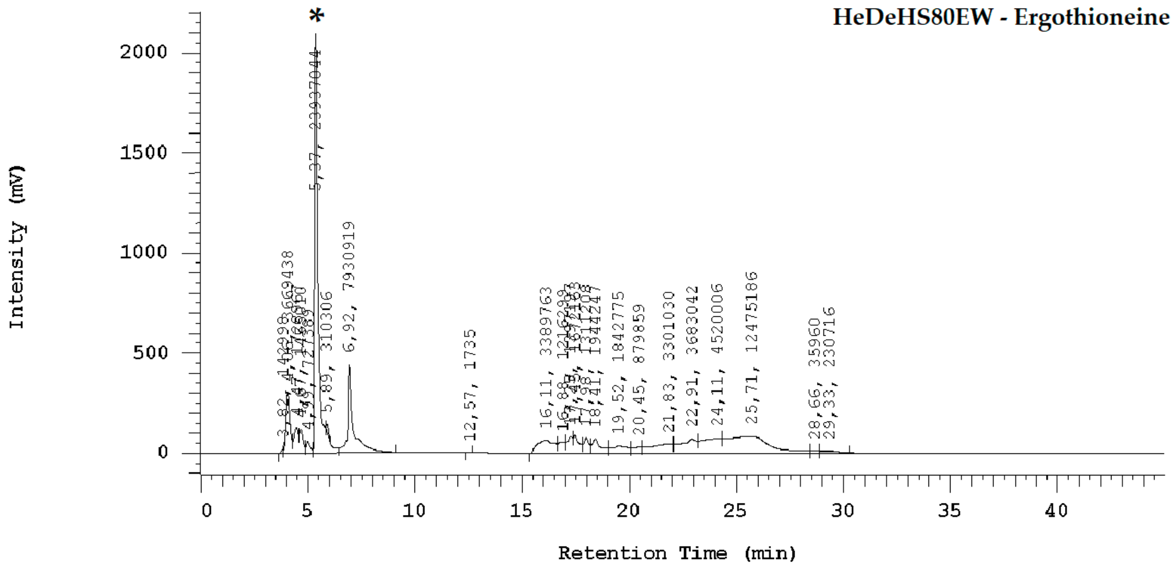

Figure S9. Chromatogram of the separation of ergothioneine for HeDeHS80EW.

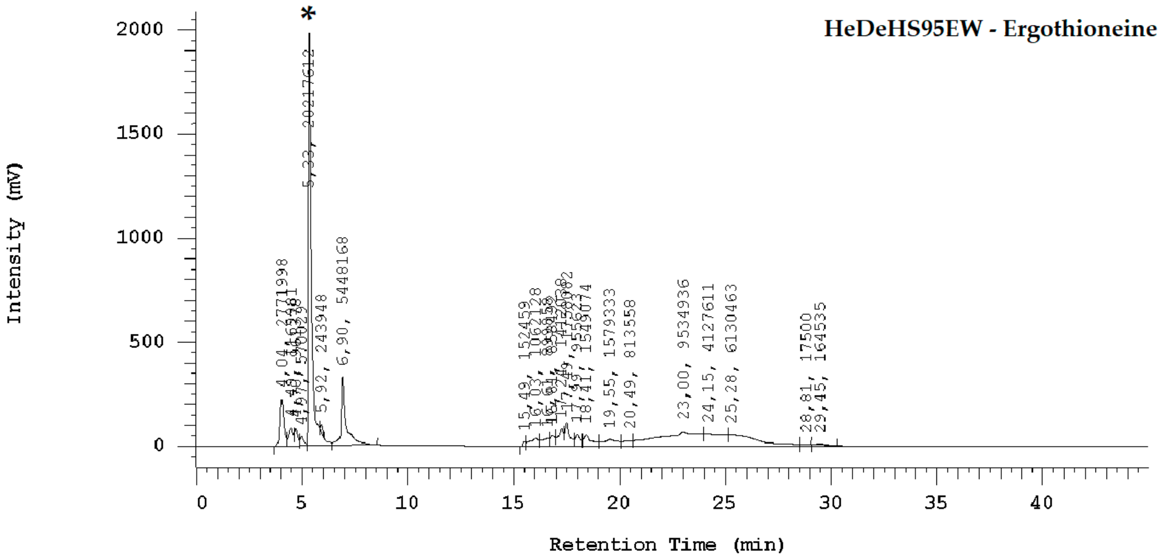

Figure S10. Chromatogram of the separation of ergothioneine for HeDeHS95EW.

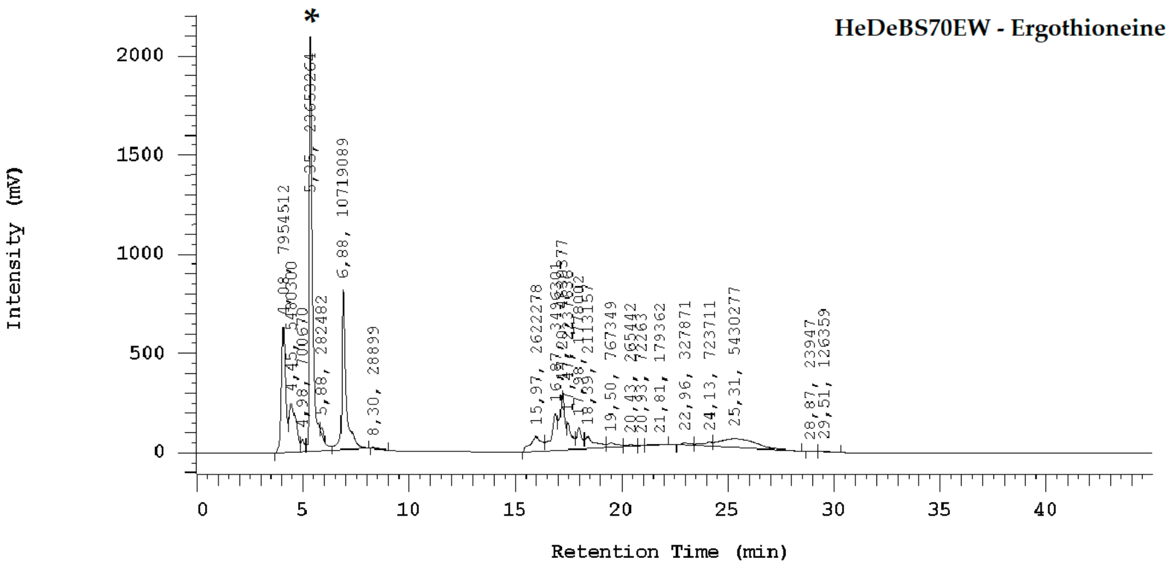

Figure S11. Chromatogram of the separation of ergothioneine for HeDeBS70EW.

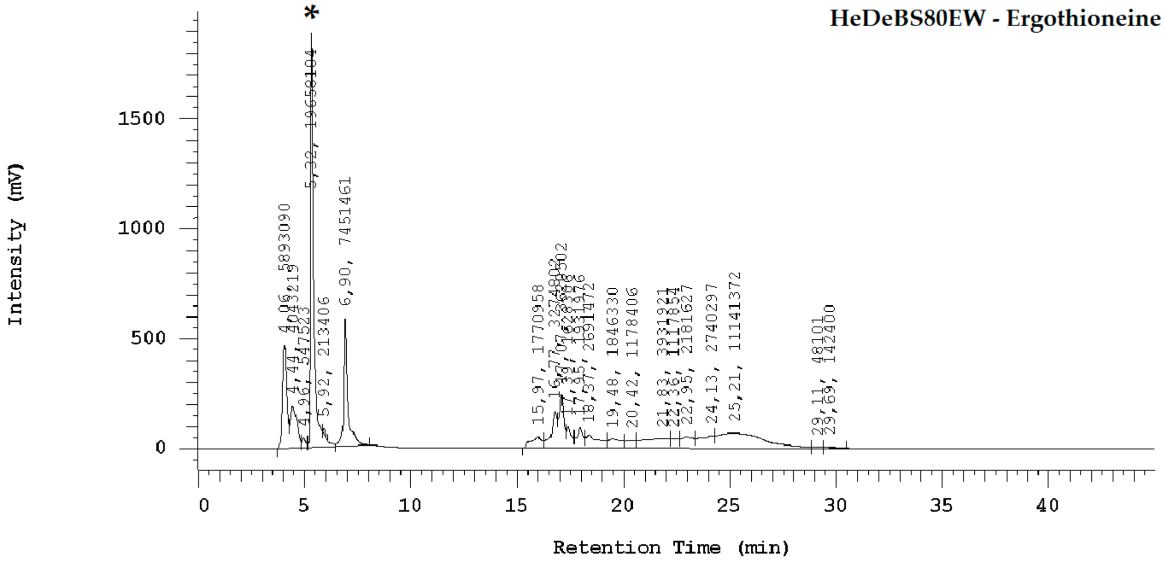

Figure S12. Chromatogram of the separation of ergothioneine for HeDeBS80EW.

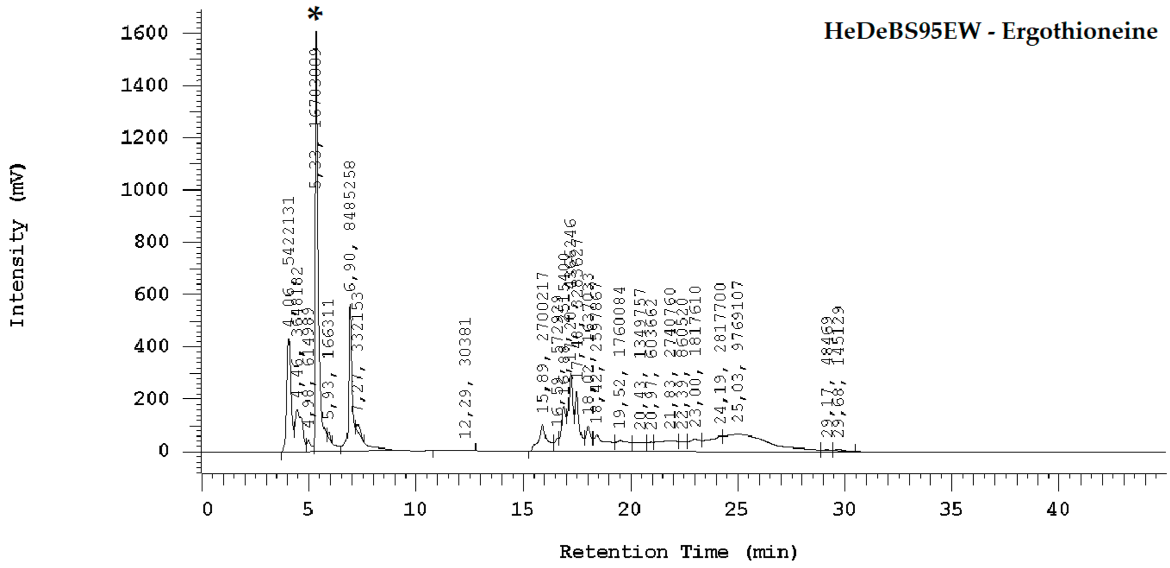

Figure S13. Chromatogram of the separation of ergothioneine for HeDeBS95EW.

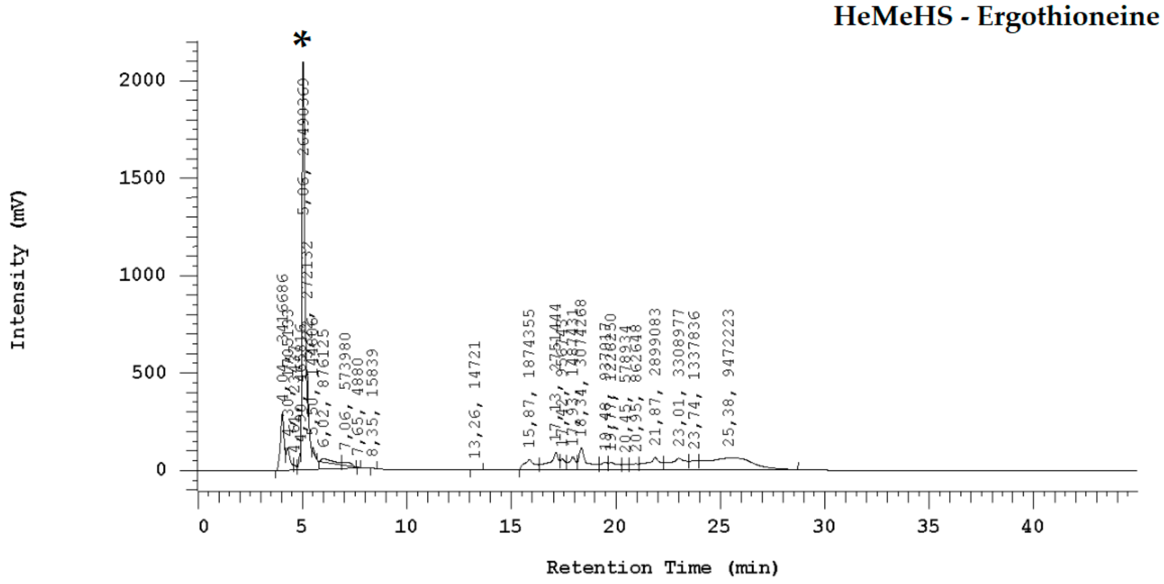

Figure S14. Chromatogram of the separation of ergothioneine for HeMeHS.

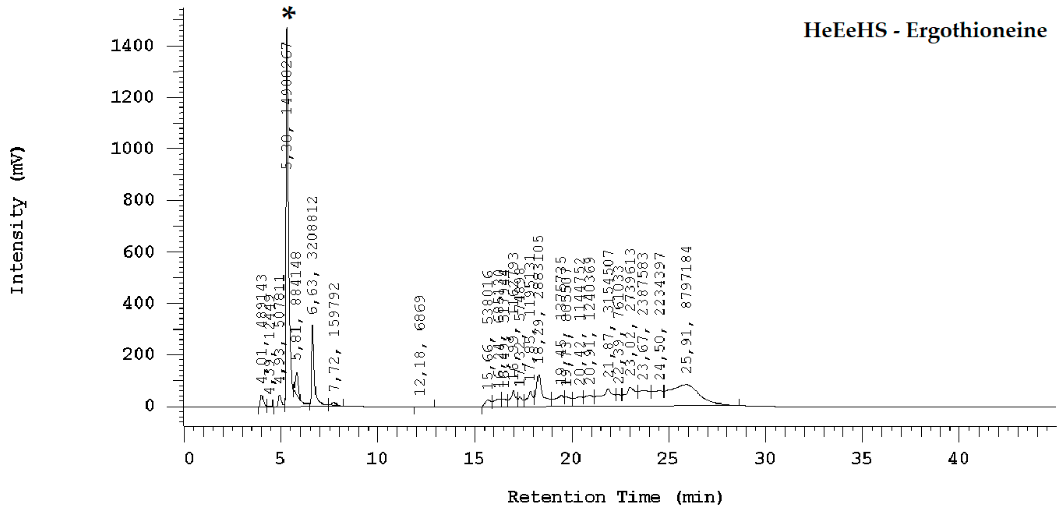

Figure S15. Chromatogram of the separation of ergothioneine for HeEeHS.

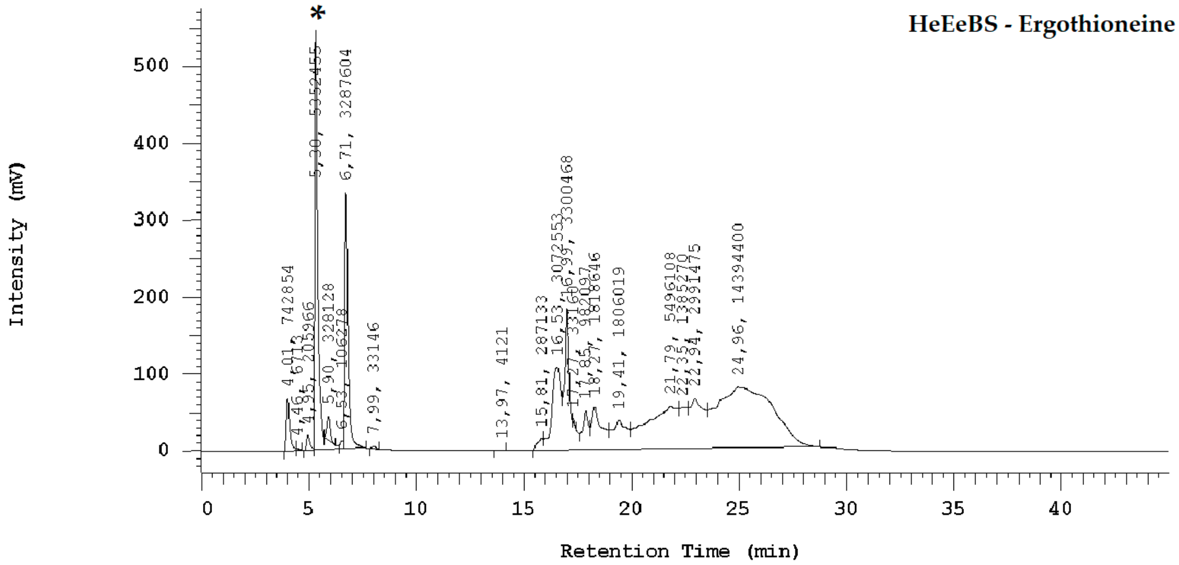

Figure S16. Chromatogram of the separation of ergothioneine for HeEeBs.

Chromatograms for the analyses lovastatin:

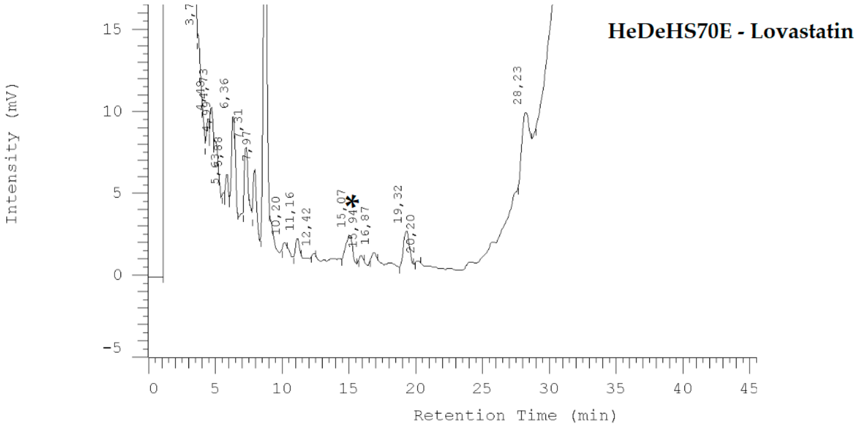

Figure S17. Chromatogram of the separation of lovastatin for HeDeHS70E.

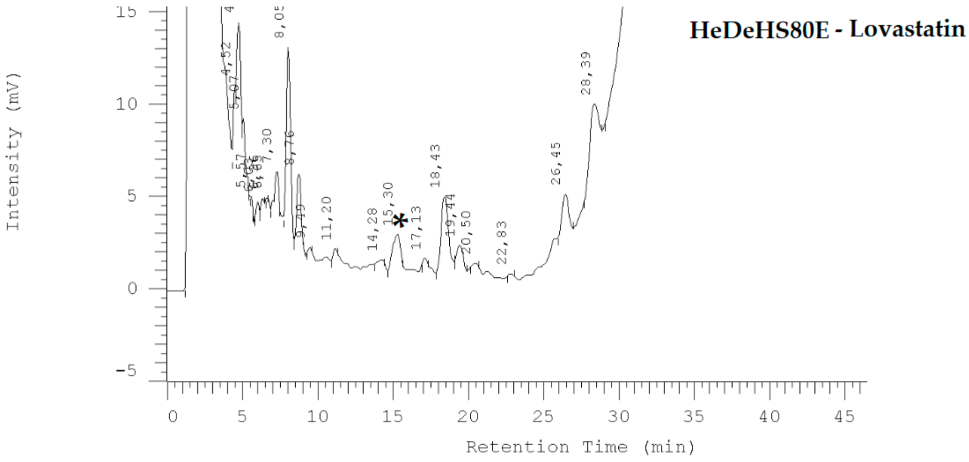

Figure S18. Chromatogram of the separation of lovastatin for HeDeHS80E.

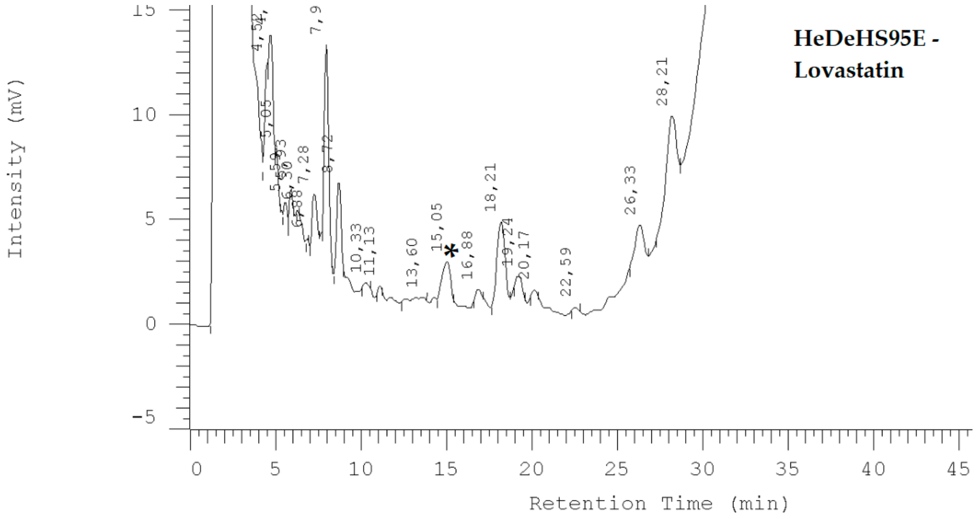

Figure S19. Chromatogram of the separation of lovastatin for HeDeHS95E.

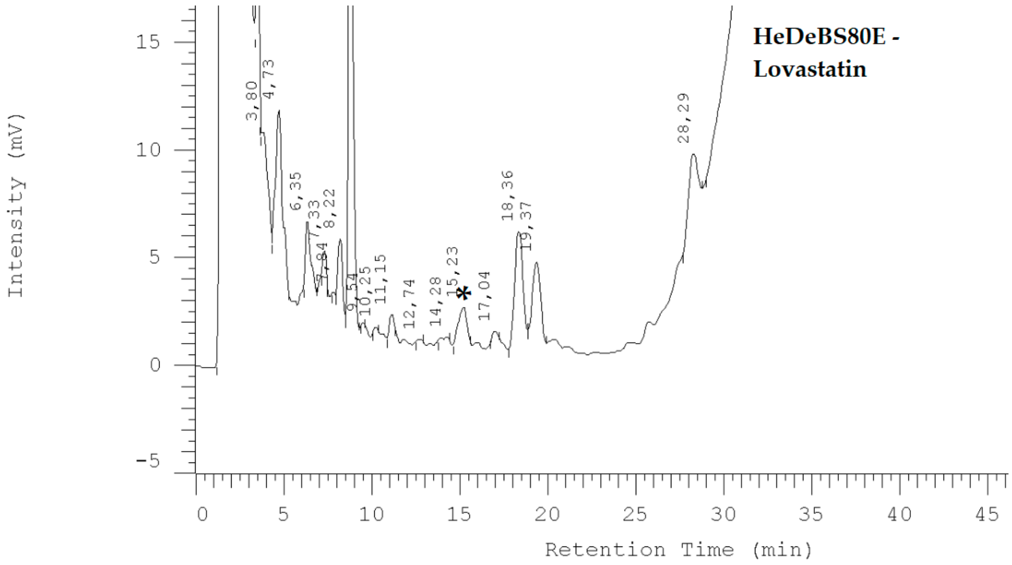

Figure S20. Chromatogram of the separation of lovastatin for HeDeBS80E.

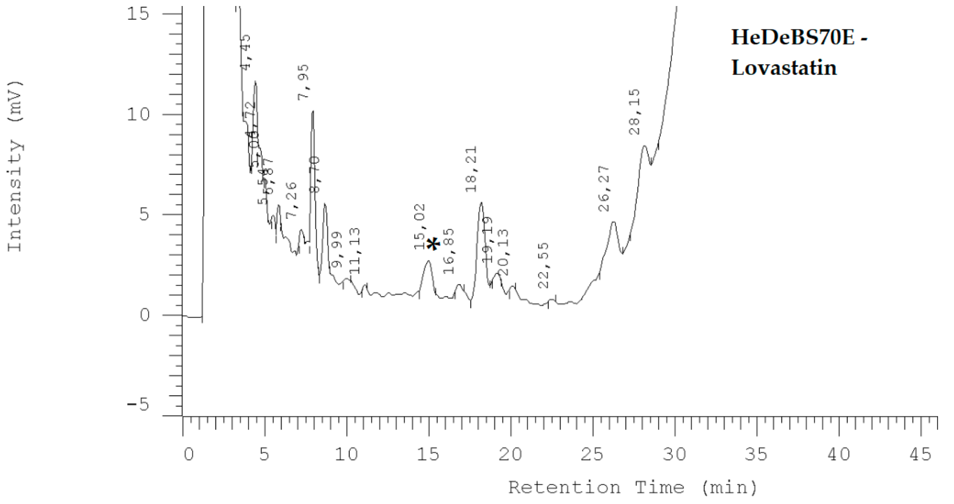

Figure S21. Chromatogram of the separation of lovastatin for HeDeBS70E.

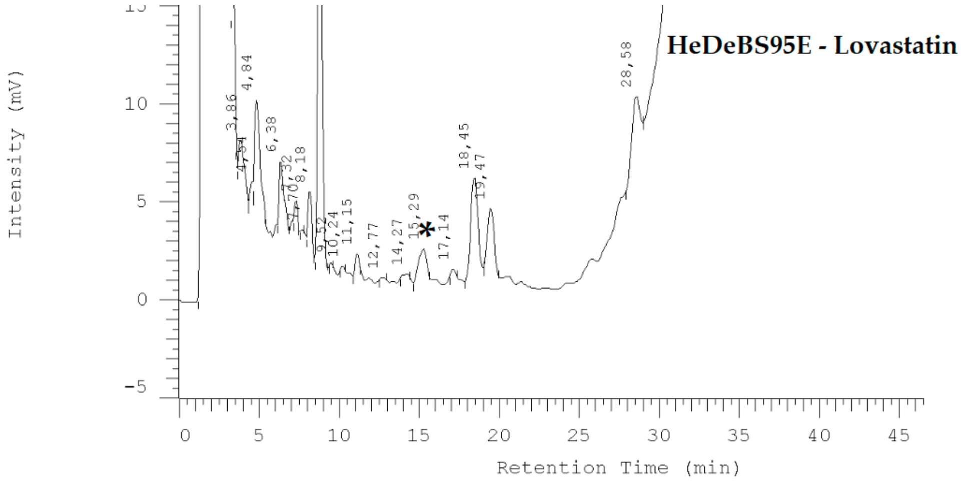

Figure S22. Chromatogram of the separation of lovastatin for HeDeBS95E.

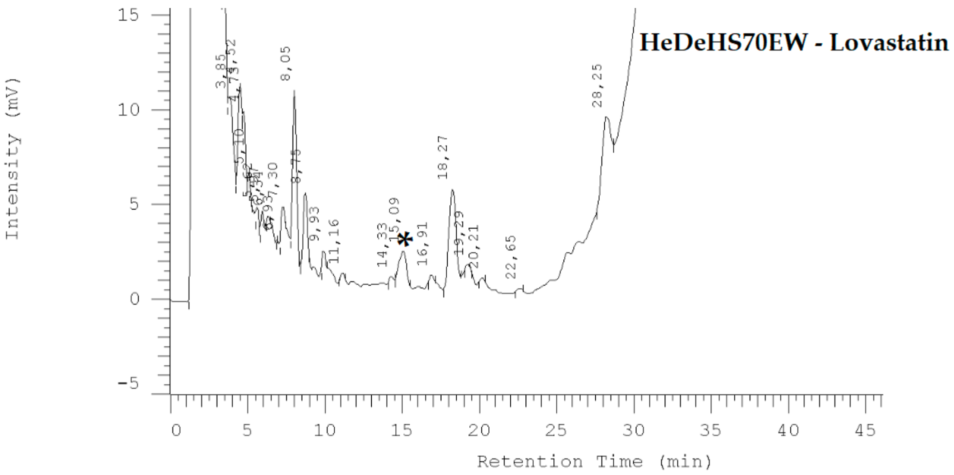

Figure S23. Chromatogram of the separation of lovastatin for HeDeHS70EW.

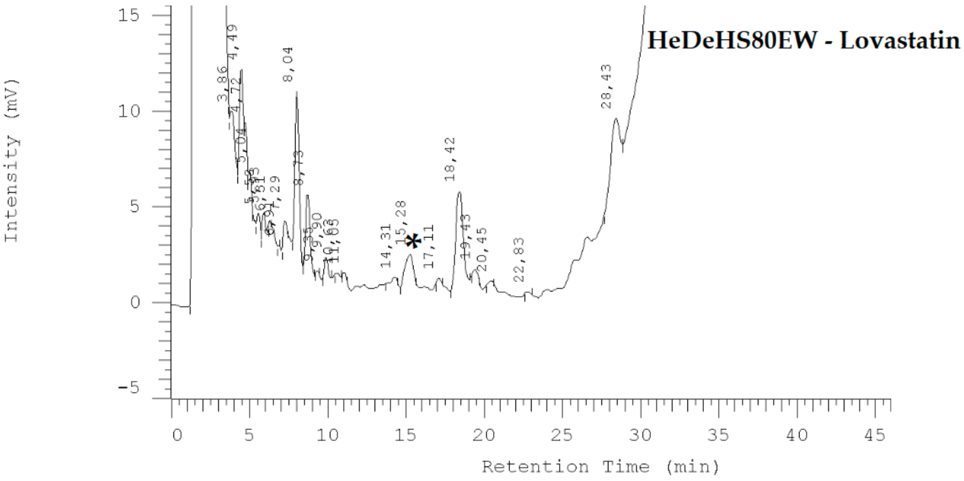

Figure S24. Chromatogram of the separation of **lovastatin** for HeDeHS80EW.

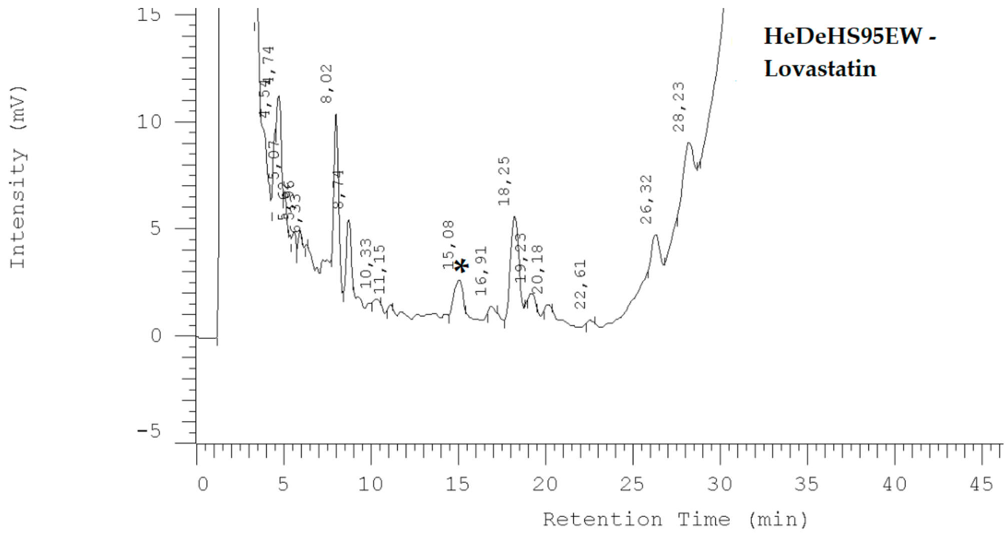

Figure S25. Chromatogram of the separation of lovastatin for HeDeHS95EW.

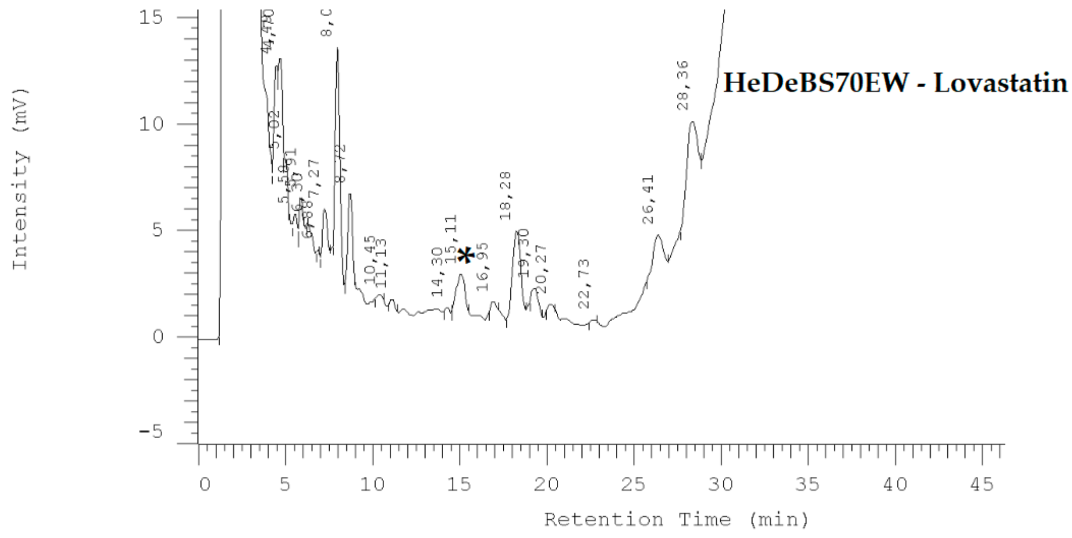

Figure S26. Chromatogram of the separation of lovastatin for HeDeBS70EW.

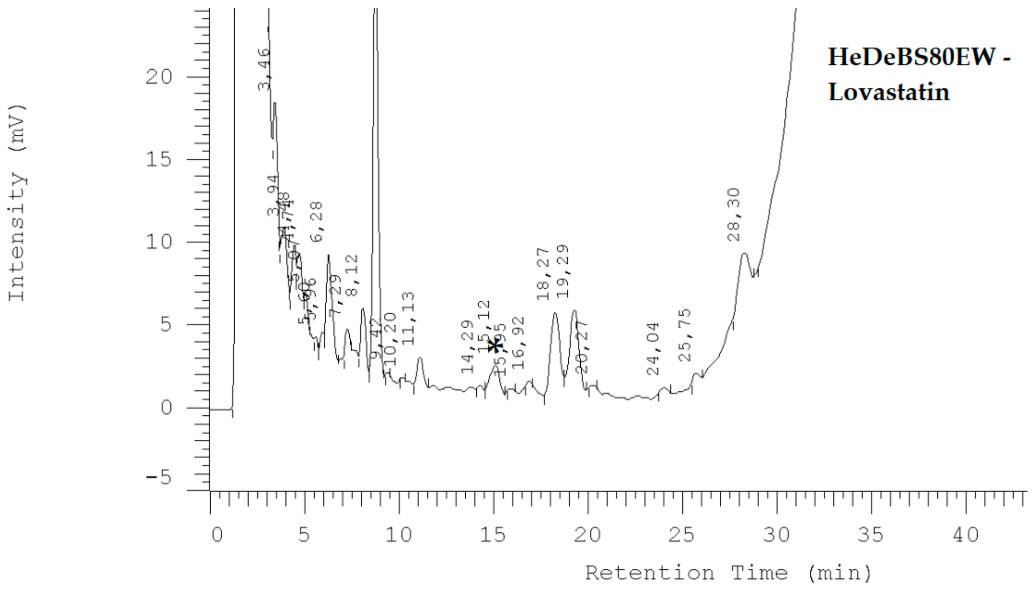

Figure S27. Chromatogram of the separation of lovastatin for HeDeBS80EW.

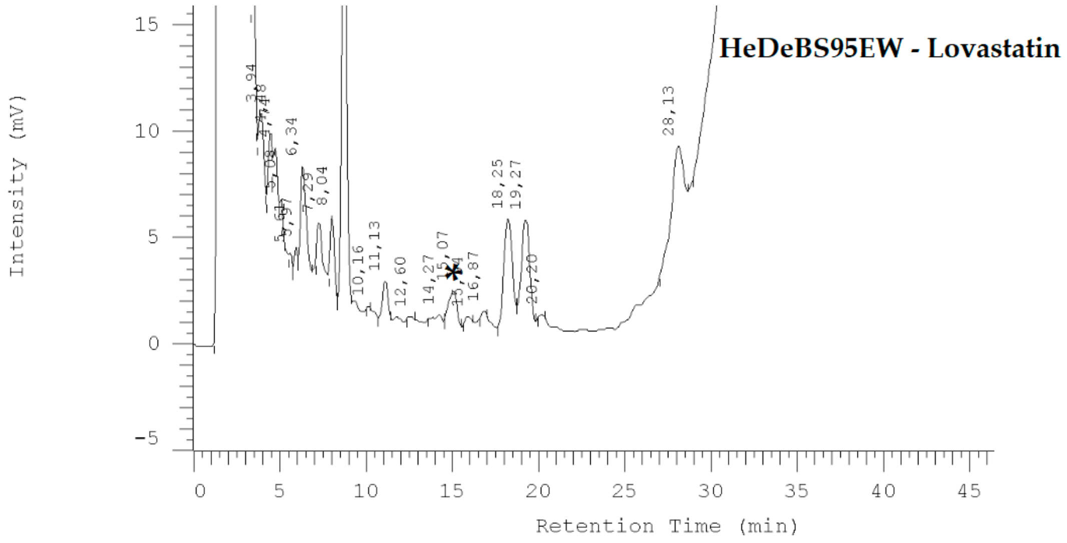

Figure S28. Chromatogram of the separation of lovastatin for HeDeBS95EW.

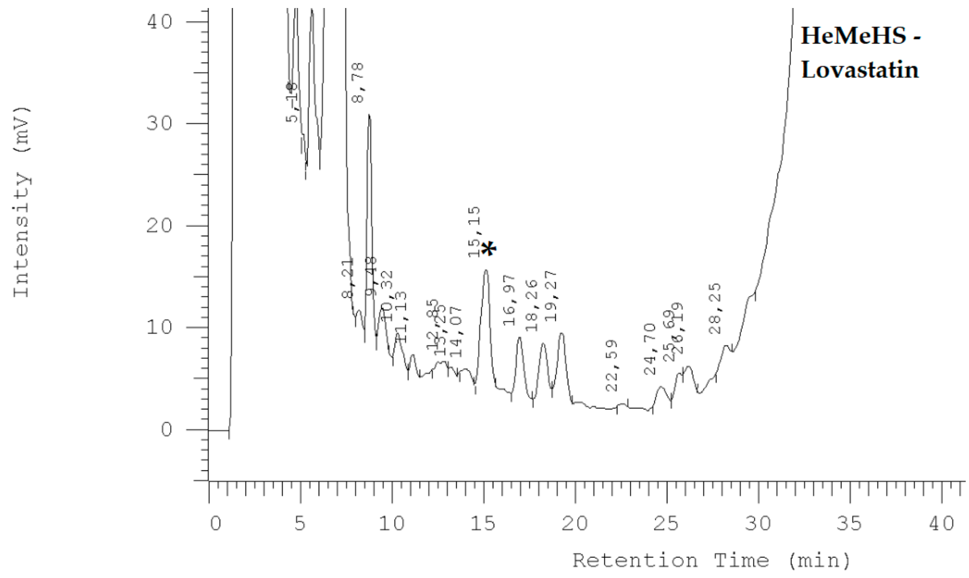

Figure S29. Chromatogram of the separation of lovastatin for HeMeHS.

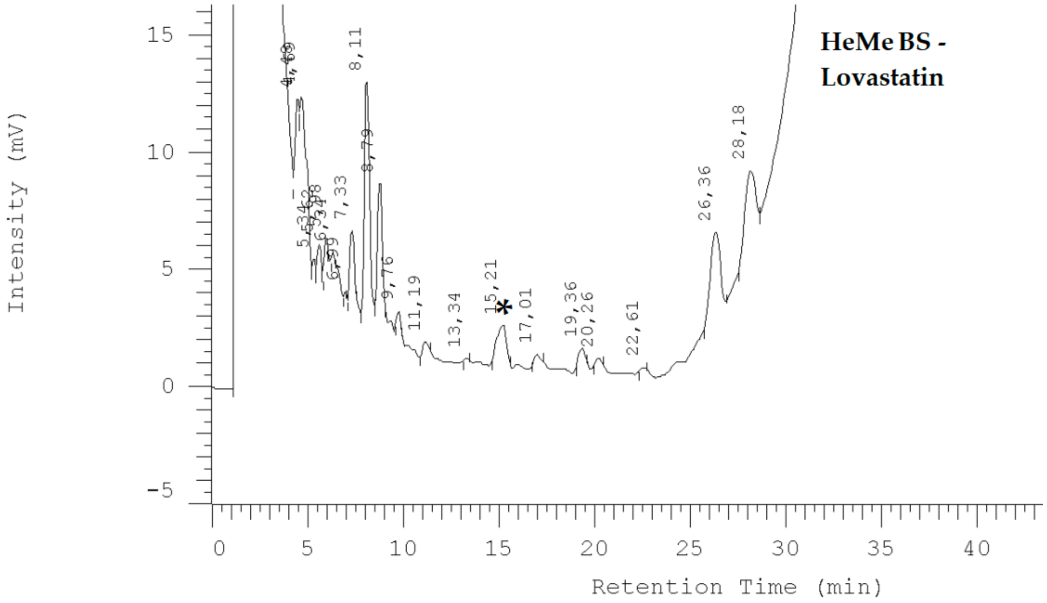

Figure S30. Chromatogram of the separation of lovastatin for HeMeBS.

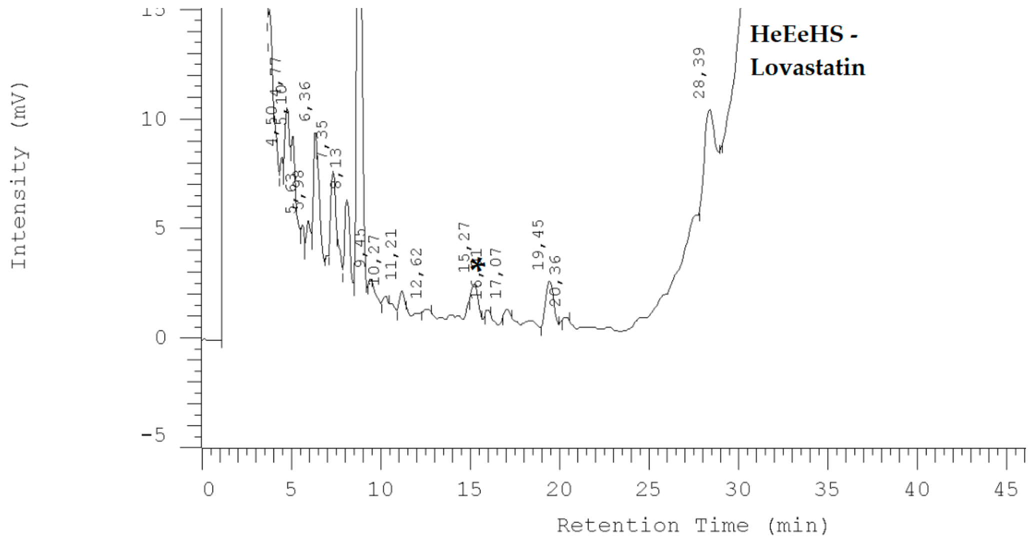

Figure S31. Chromatogram of the separation of lovastatin for HeEeHs.

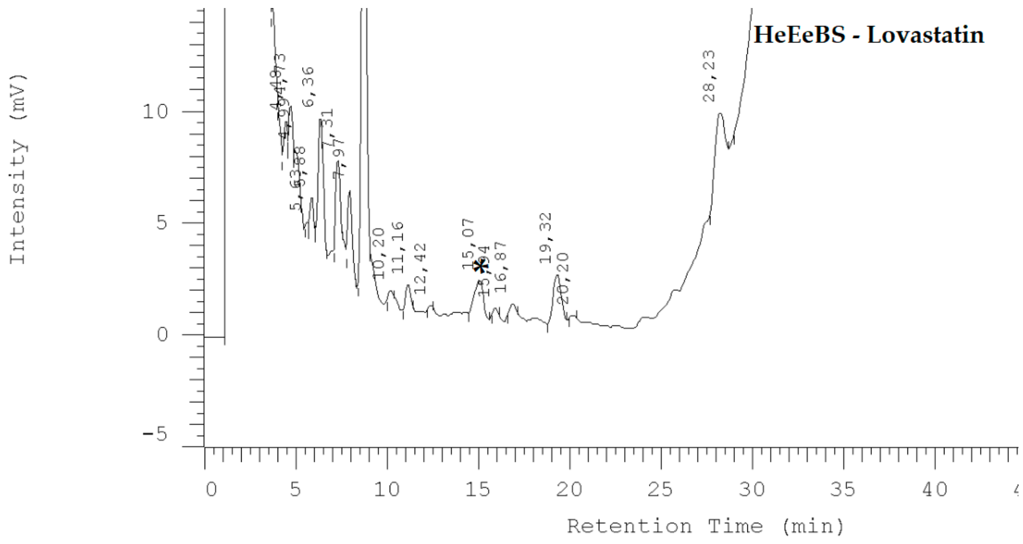

Figure S32. Chromatogram of the separation of lovastatin for HeEeBs.

Chromatograms for the analysis of serotonin:

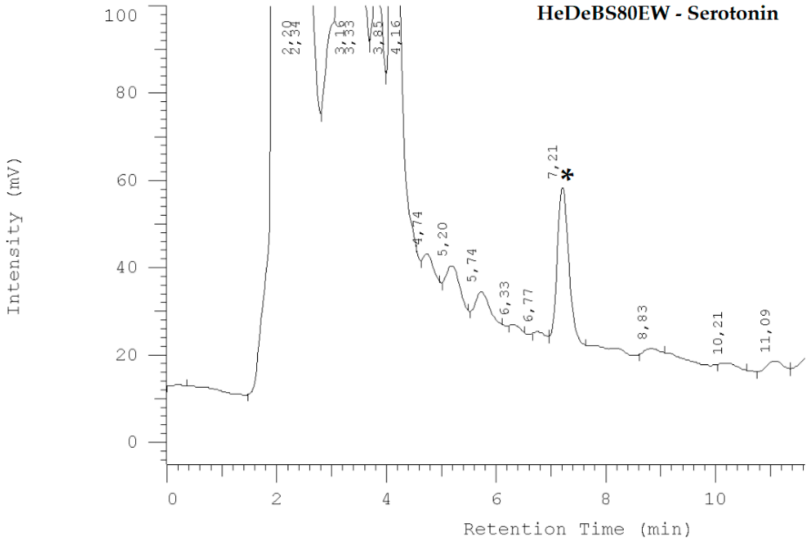

Figure S32. Chromatogram of the separation of serotonin for HeDeBS80EW.

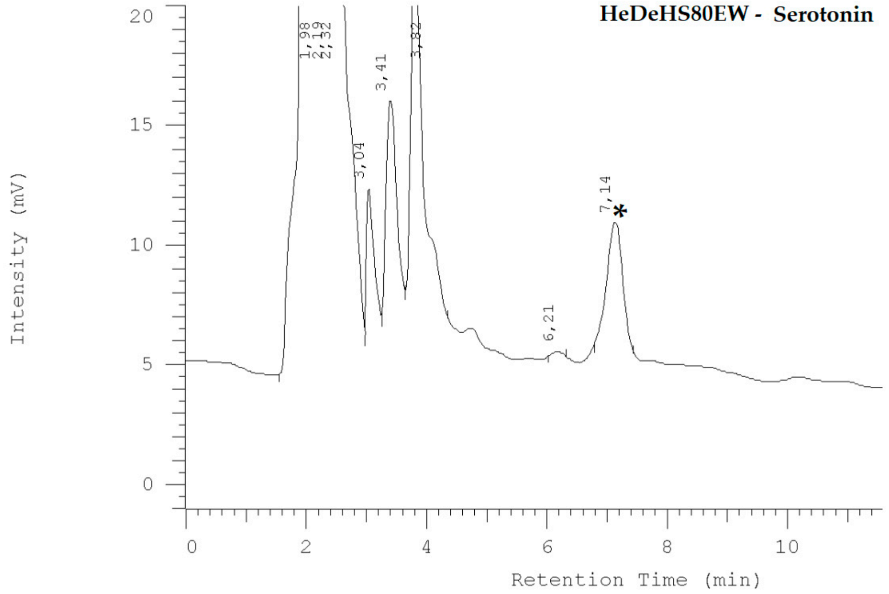

Figure S34. Chromatogram of the separation of serotonin for HeDeHS80EW.

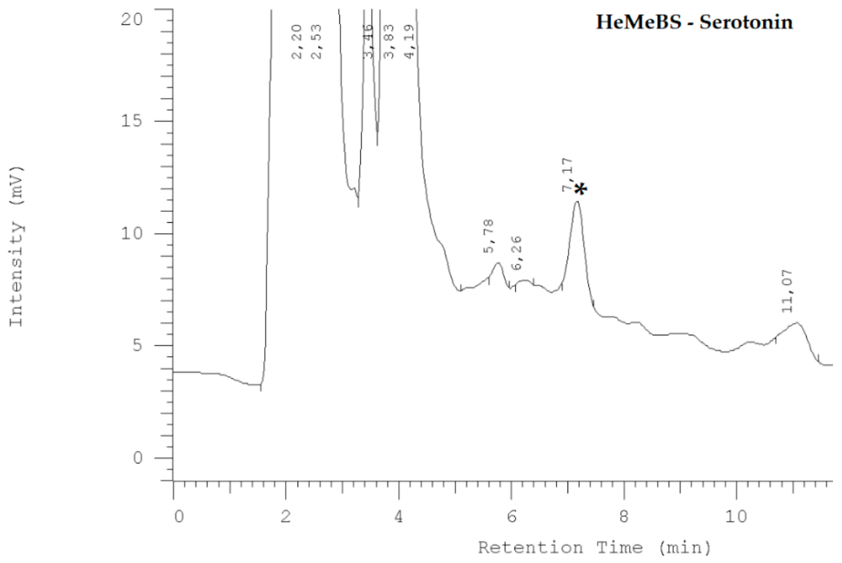

Figure S35. Chromatogram of the separation of serotonin for HeMeBS.

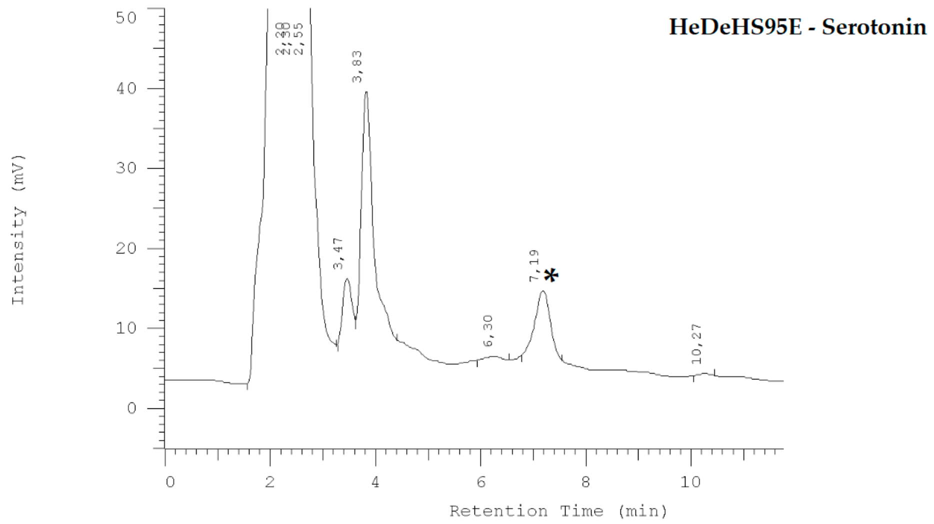

Figure S36. Chromatogram of the separation of serotonin for HeDeHS95E.

Chromatograms for the analysis of L-phenylalanine:

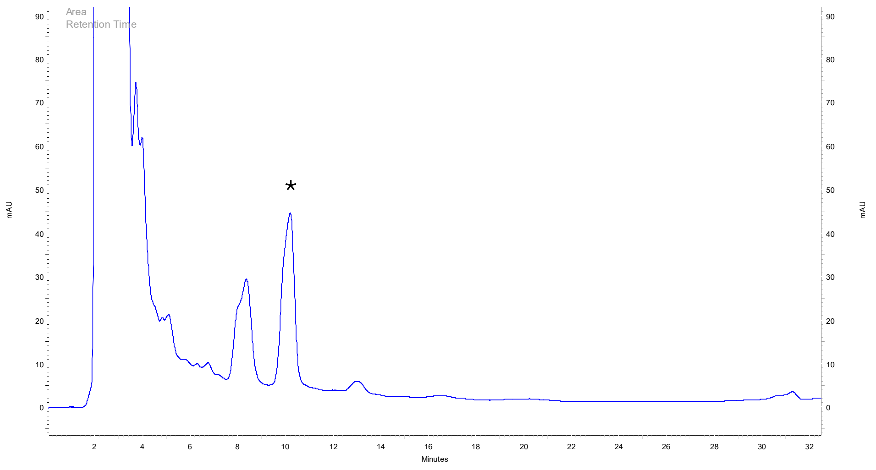

Figure S37. Chromatogram of the separation of L-phenylalanine for HeDeHS70E.

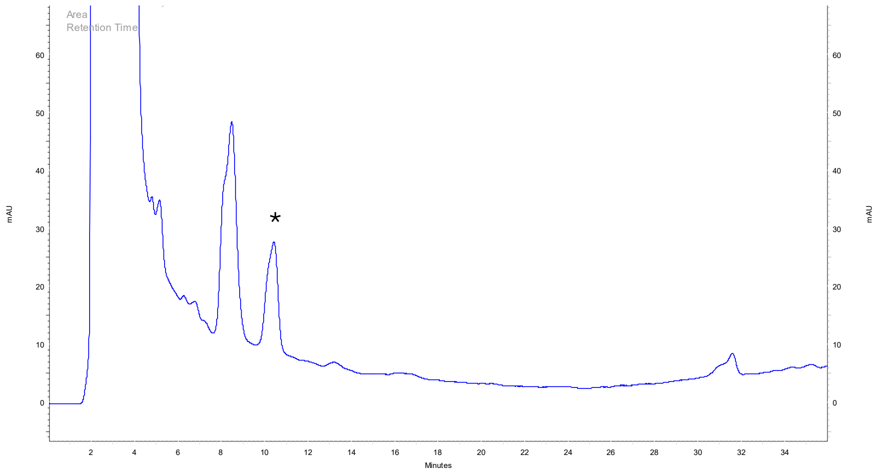

Figure S38. Chromatogram of the separation of L-phenylalanine for HeDeHS80E.

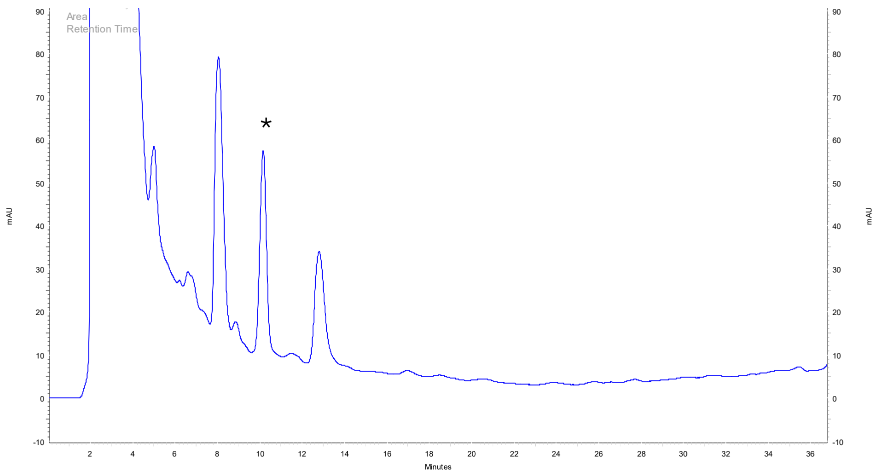

Figure S39. Chromatogram of the separation of L-phenylalanine for HeDeHS95E.

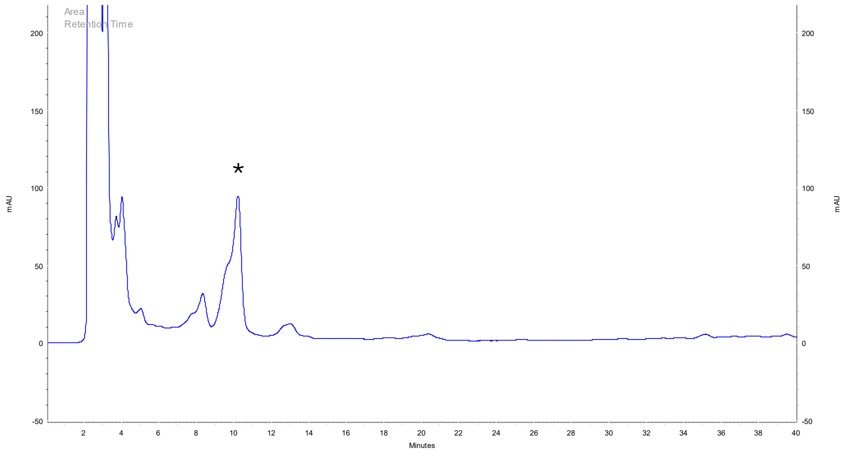

Figure S40. Chromatogram of the separation of L-phenylalanine forHeDeBS70E.

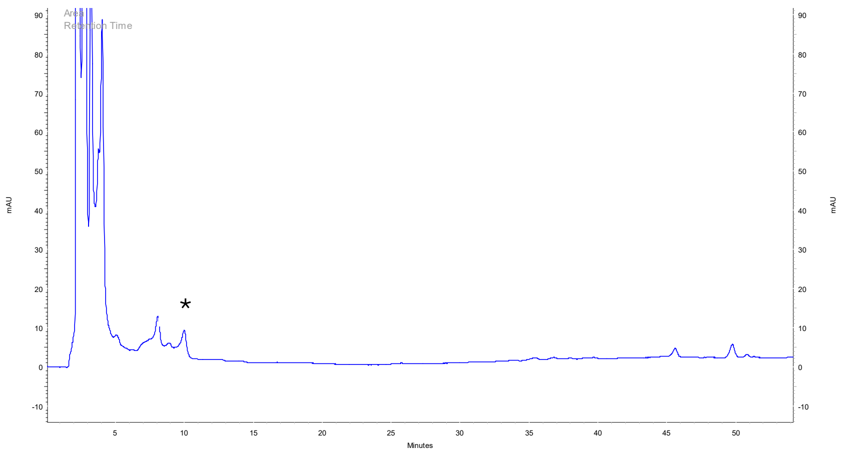

Figure S41. Chromatogram of the separation of L-phenylalanine forHeDeBS80E.

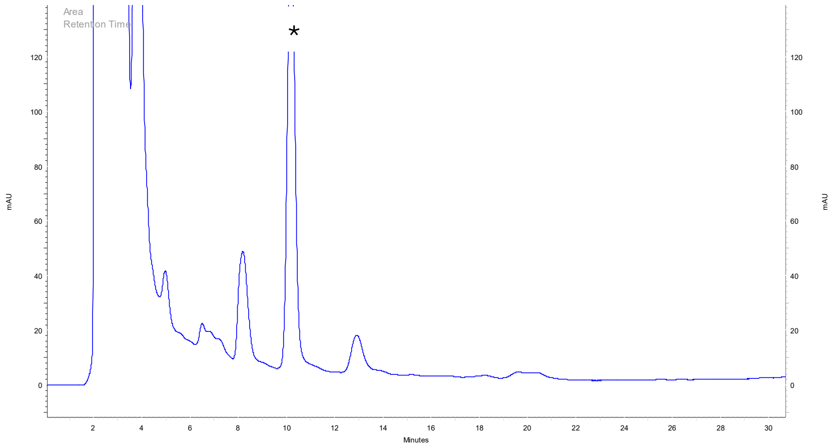

Figure S42. Chromatogram of the separation of L-phenylalanine forHeDeBS95E.

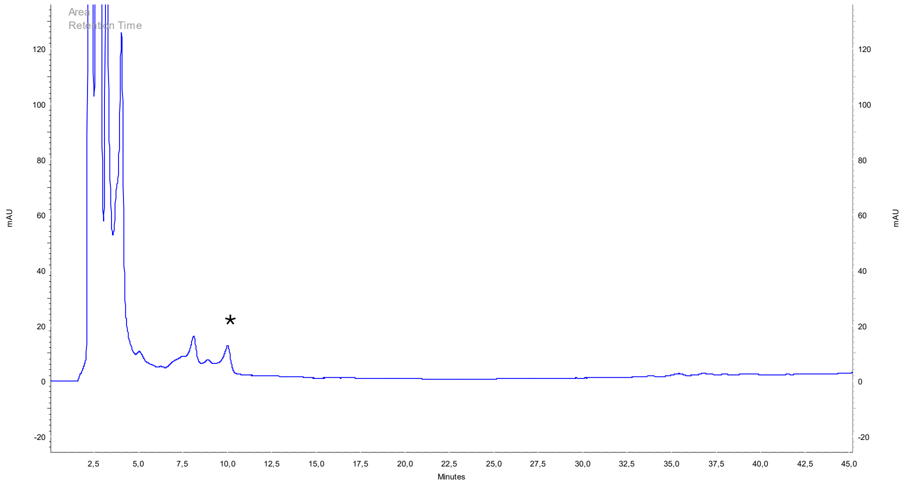

Figure S43. Chromatogram of the separation of L-phenylalanine for HeDeHS70EW.

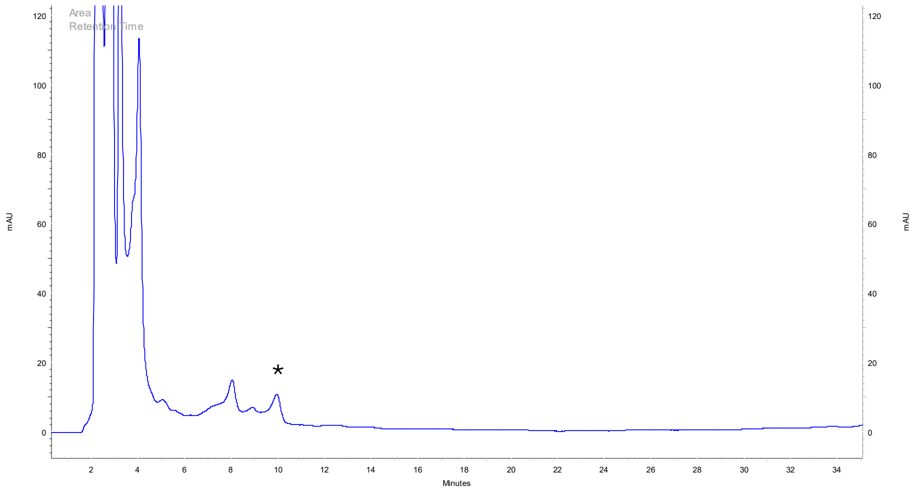

Figure S44. Chromatogram of the separation of L-phenylalanine for HeDeHS80EW.

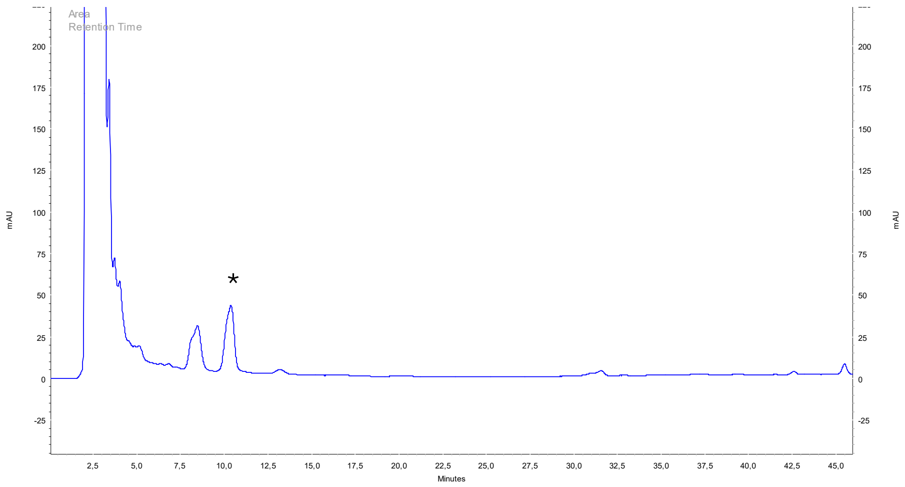

Figure S45. Chromatogram of the separation of L-phenylalanine for HeDeHS95EW.

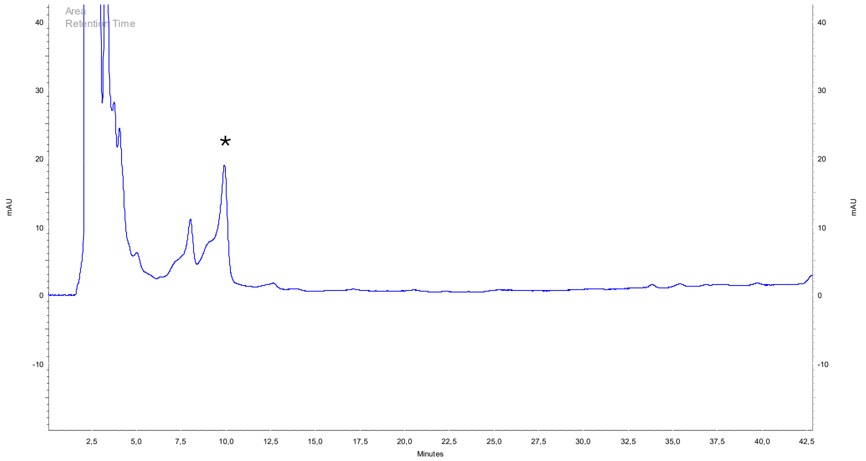

Figure S46. Chromatogram of the separation of L-phenylalanine e for HeDeBS70EW .

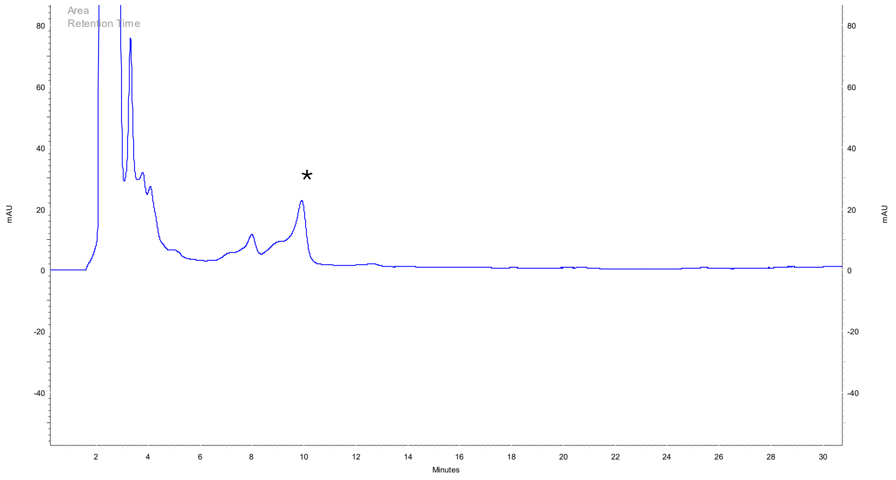

Figure S47. Chromatogram of the separation of L-phenylalanine for HeDeBS80EW.

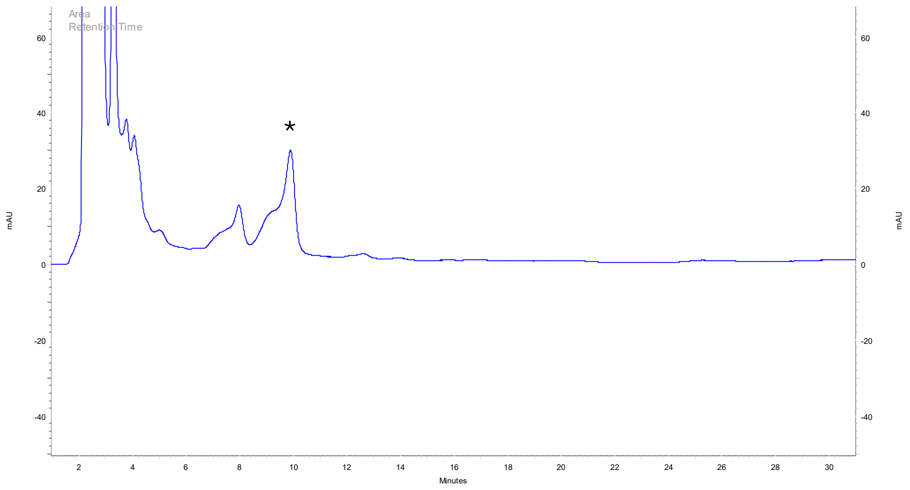

Figure S48. Chromatogram of the separation of L-phenylalanine for HeDeBS95EW.

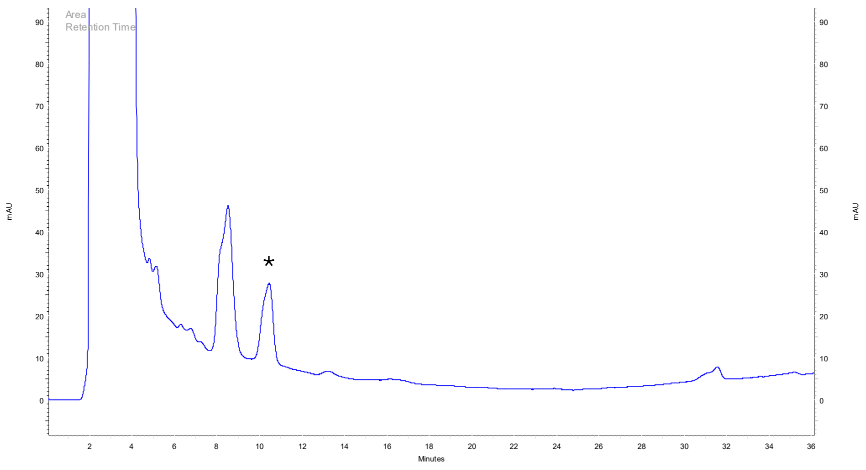

Figure S49. Chromatogram of the separation of L-phenylalanine e for HeMeHS.

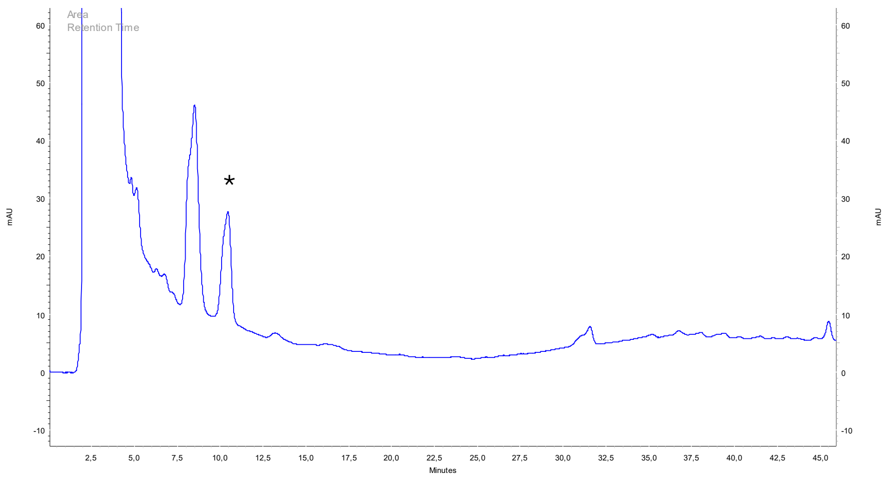

Figure S50. Chromatogram of the separation of L-phenylalanine for HeMeBS .

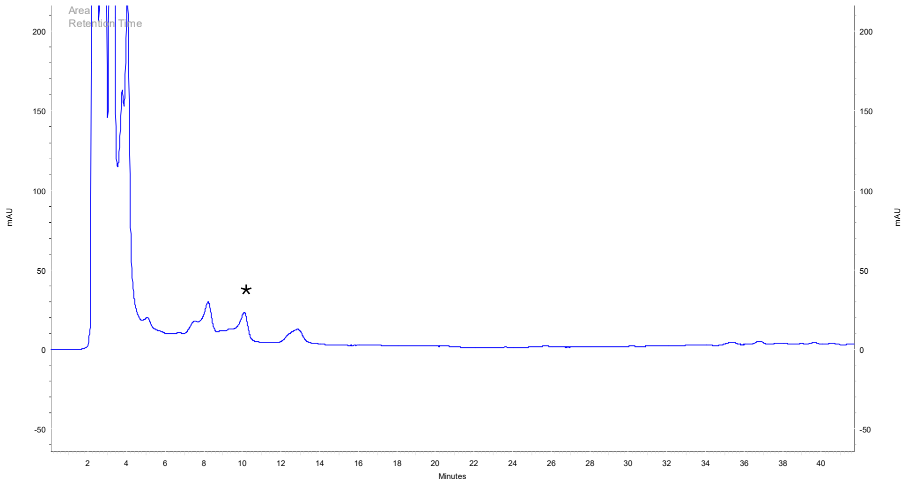

Figure S51. Chromatogram of the separation of L-phenylalanine e for HeEeHS.

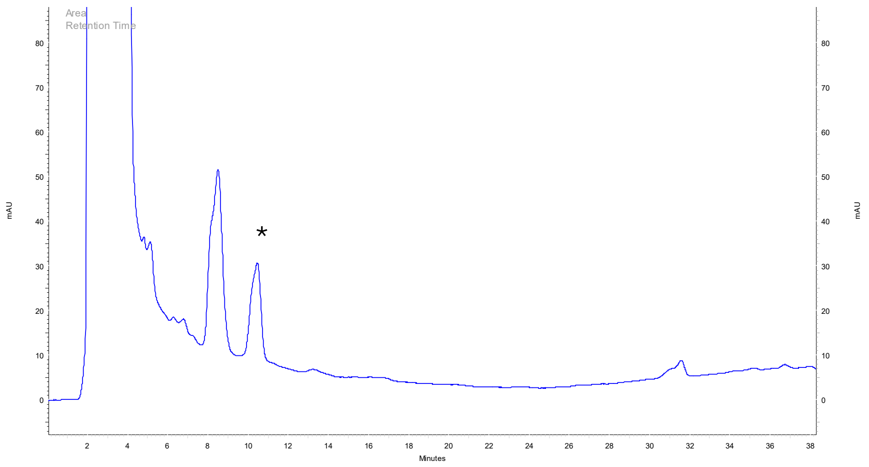

Figure S52. Chromatogram of the separation of L-phenylalanine for HeEeBS.

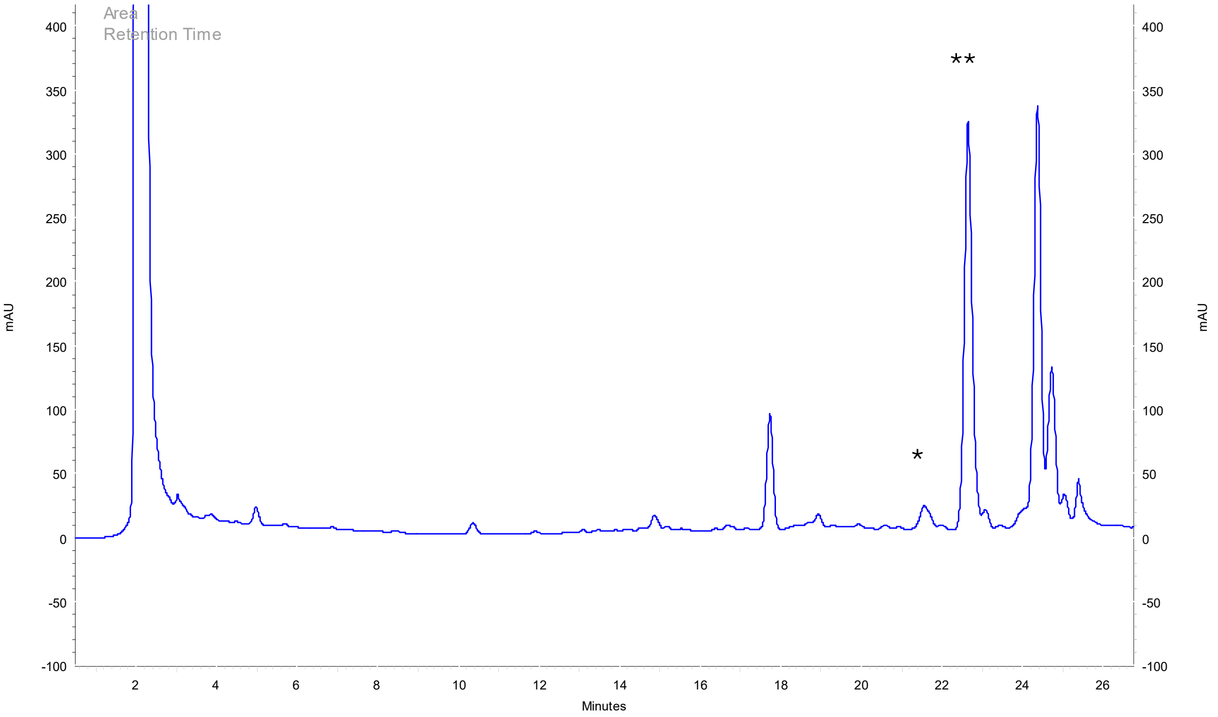

Figure S53. Chromatogram of the separation of tocopherol (*) and ergosterol (**) for HeDeHS70E.

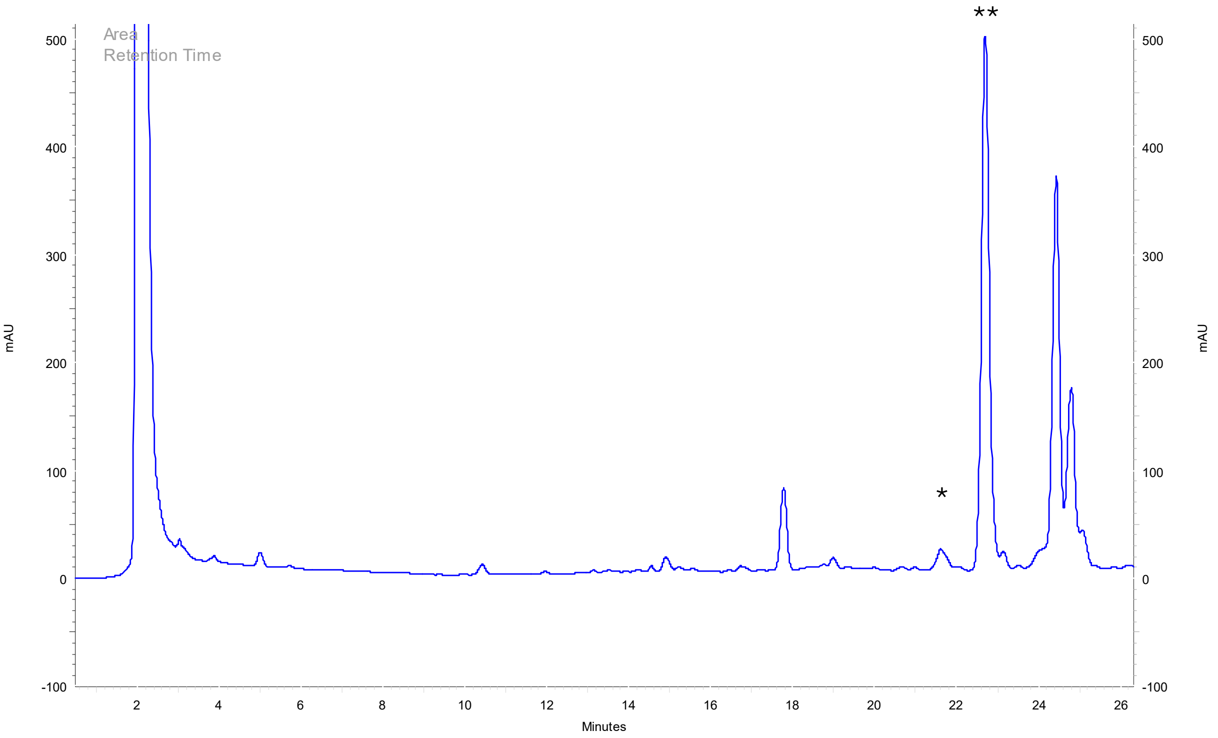

Figure S54. Chromatogram of the separation of tocopherol (*) and ergosterol (**) for HeDeHS80E.

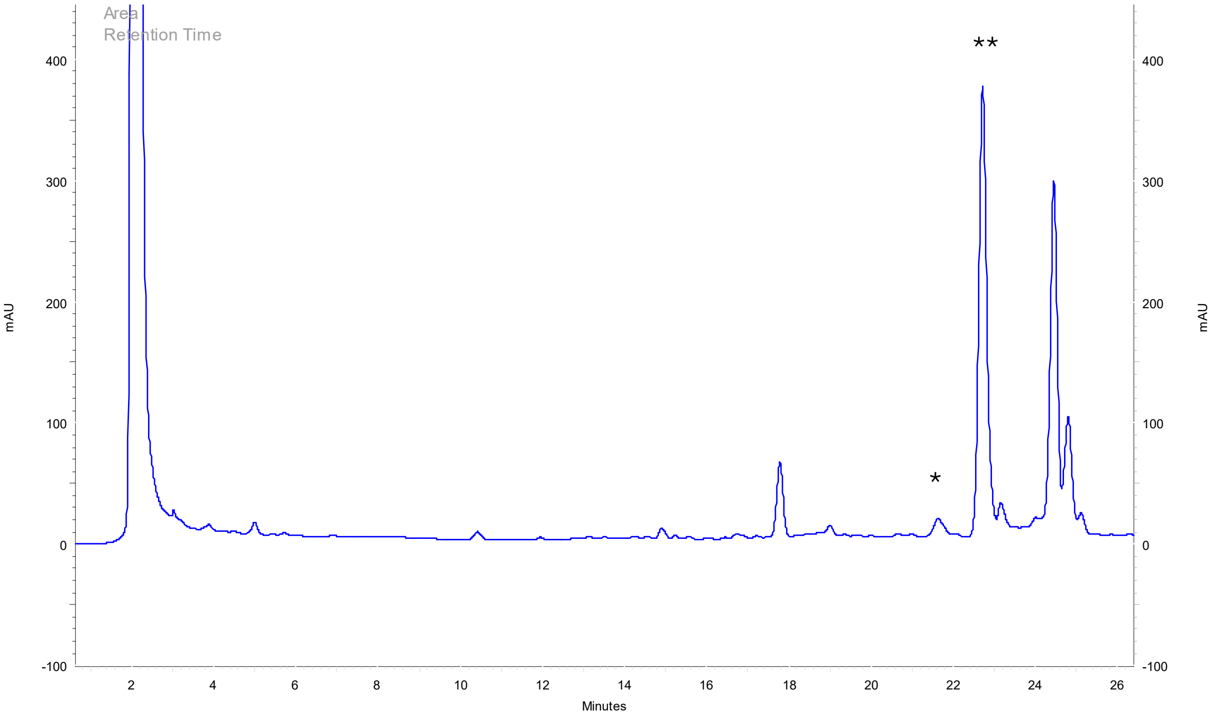

Figure S55. Chromatogram of the separation of tocopherol (*) and ergosterol (**) for HeDeHS95E.

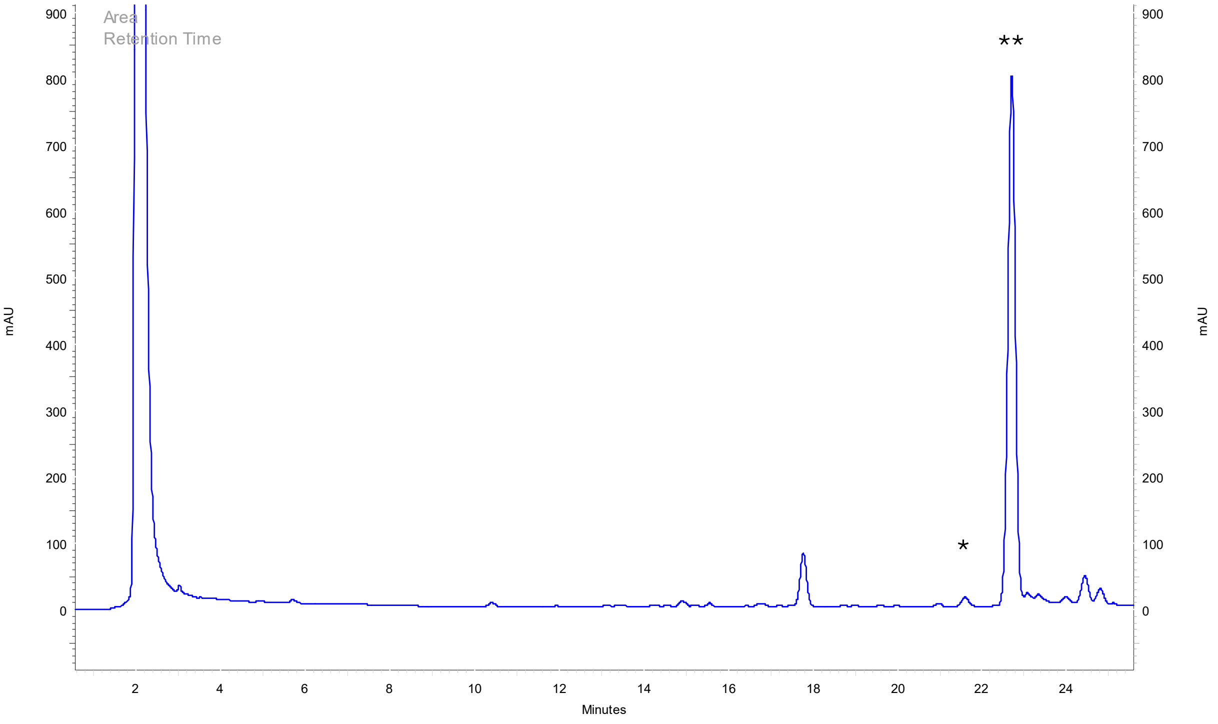

Figure S56. Chromatogram of the separation of tocopherol (*) and ergosterol (**) for HeDeBS70E.

*

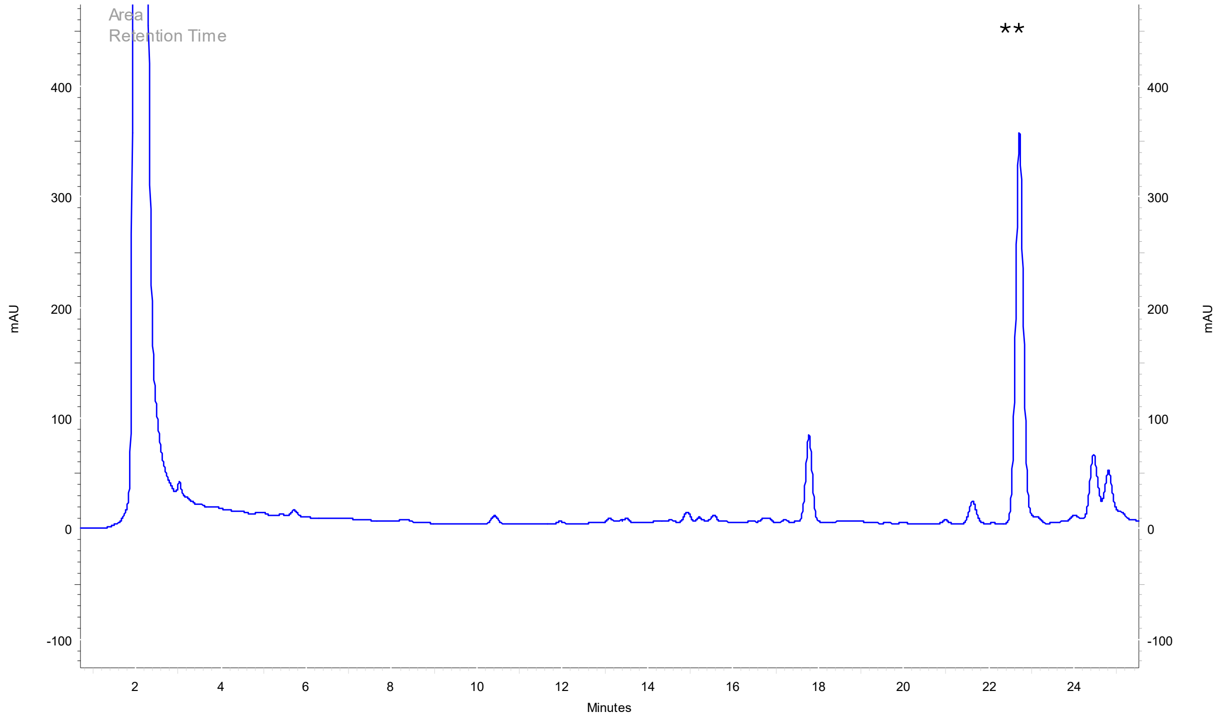

Figure S57. Chromatogram of the separation of tocopherol (*) and ergosterol (**) for HeDeBS70E.

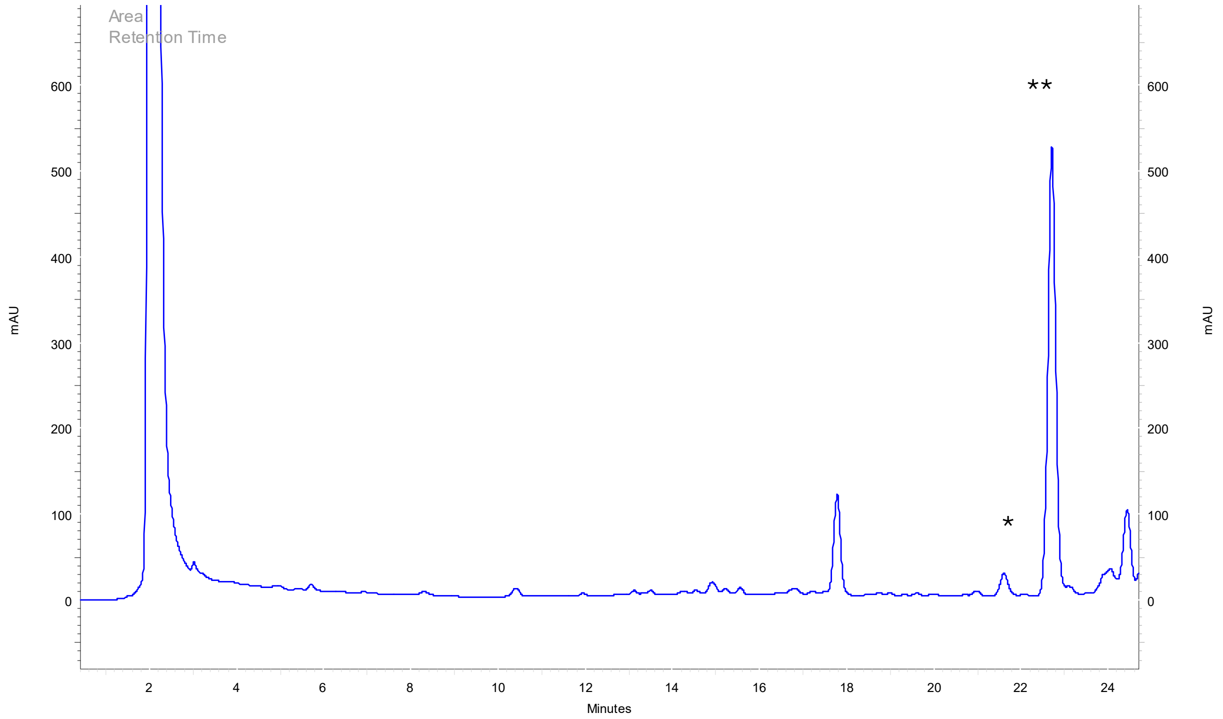

Figure S58. Chromatogram of the separation of tocopherol (*) and ergosterol (**) for HeDeBS70E.

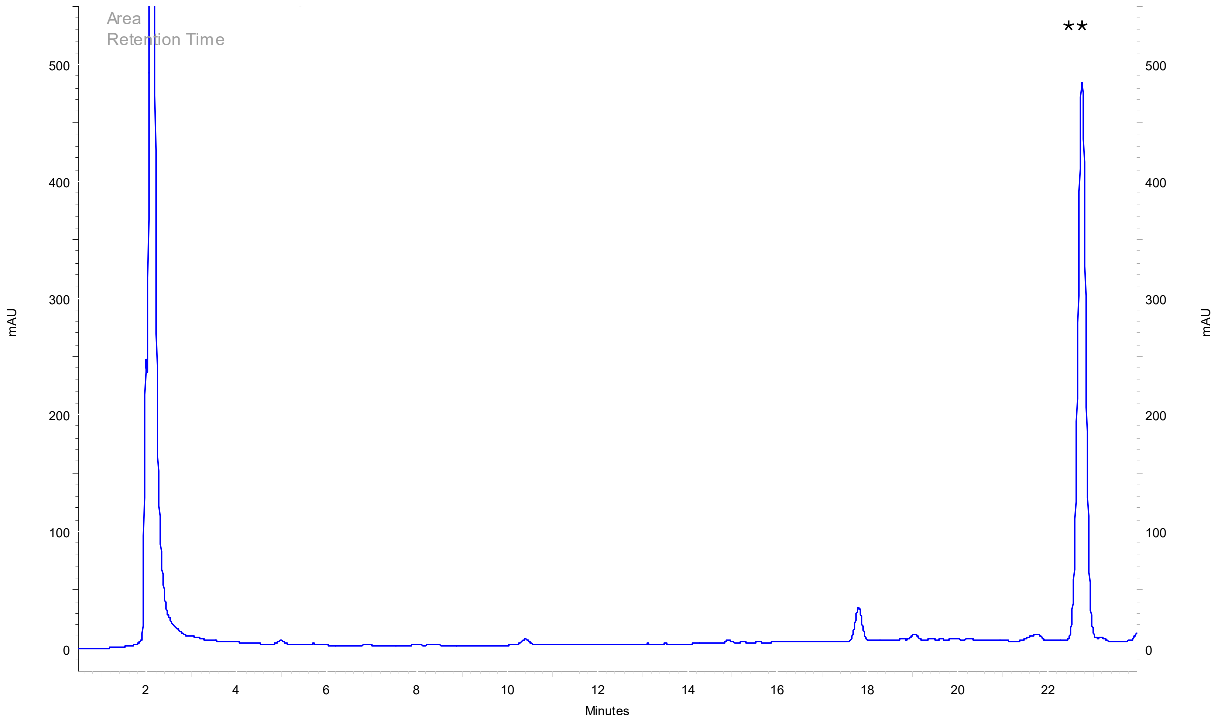

Figure S59. Chromatogram of the separation of tocopherol (*) and ergosterol (**) for HEFbHS.

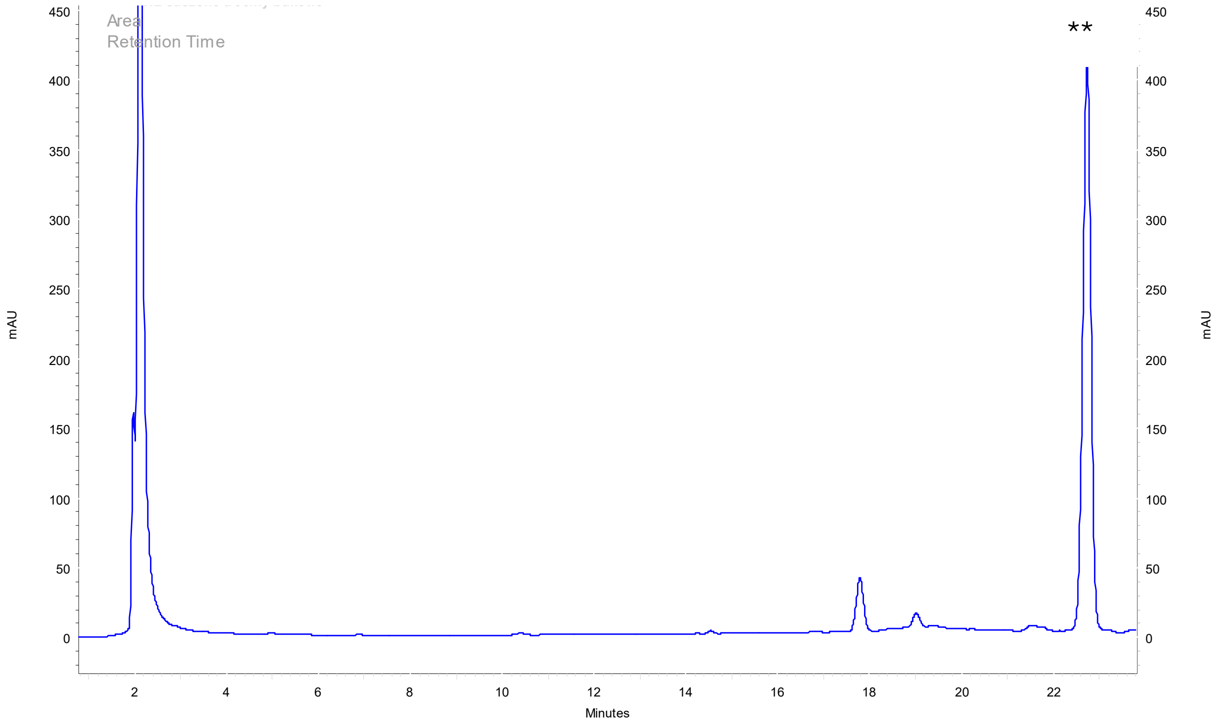

Figure S60. Chromatogram of the separation of tocopherol (*) and ergosterol (**) for HEFbBS.
